# The interferon-β/STAT1 axis drives the collective invasion of skin squamous cell carcinoma with sealed intercellular spaces

**DOI:** 10.1038/s41389-022-00403-9

**Published:** 2022-05-24

**Authors:** Yuji Kumagai, Junko Nio-Kobayashi, Seiichiro Ishihara, Atsushi Enomoto, Masashi Akiyama, Ryosuke Ichihara, Hisashi Haga

**Affiliations:** 1 Division of Life Science, Graduate School of Life Science, Hokkaido University, N10-W8, Kita-ku, Sapporo, 060-0810 Japan; 2 Laboratory of Histology and Cytology, Faculty of Medicine and Graduate School of Medicine, Hokkaido University, N15-W7, Kita-ku, Sapporo, 060-8638 Japan; 3 Department of Advanced Transdisciplinary Sciences, Faculty of Advanced Life Science, Hokkaido University, N10-W8, Kita-ku, Sapporo, 060-0810 Japan; 4 Department of Pathology, Graduate School of Medicine, Nagoya University, 65 Tsurumai-cho, Showa-ku, Nagoya, 466-8550 Japan; 5 Department of Dermatology, Nagoya University Graduate School of Medicine, 65 Tsurumai-cho, Showa-ku, Nagoya, 466-8550 Japan

**Keywords:** Cancer imaging, Squamous cell carcinoma, Metastasis, Cell migration, Cell adhesion

## Abstract

The process by which cancer cells invade as a cell cluster, known as collective invasion, is associated with metastasis and worse prognosis of cancer patients; therefore, inhibition of collective invasion is considered to improve cancer treatment. However, the cellular characteristics responsible for collective invasion remain largely unknown. Here, we successfully established subclones with various invasive potentials derived from human skin squamous carcinoma cells. The cell cluster of the highly invasive subclone had a hermetically sealed and narrow intercellular space. Interferon-β was localized to the sealed intercellular spaces, leading to collective invasion via the activation of signal transducer and activator of transcription 1 (STAT1). On the other hand, interferon-β was not localized to non-sealed and wide intercellular spaces of the cell cluster of low-invasive subclone with deficient STAT1 activity. In the mixed cell cluster of high- and low-invasive subclones, the high-invasive sub-clonal cells were located at the invasive front of the invasive protrusion, leading to collective invasion by the low-invasive sub-clonal cells. Tissue microarray analysis of human skin squamous cell carcinoma (SCC) also showed enrichment of STAT1 in the invasive front of SCCs. These findings indicate that the intercellular structure controls the potential for collective invasion via STAT1 regulation in SCC.

## Introduction

Metastasis, in which cancer cells translocate to distant organs away from the primary tumor, is an important cause of cancer-related deaths [1]. During metastasis, cancer cells undergo invasion, in which these cells disseminate into the normal surrounding tissues [2–4]. Cancer cells have a variety of invasive patterns [4, 5], and one of which, collective invasion, has recently come to light [4, 6, 7]. The process of collective invasion, during which cancer cell clusters invade the surrounding stroma and penetrate the circulatory system, such as blood and lymphatic vessels, while retaining their epithelial characteristics, has been universally observed in a variety of cancer tissues, including squamous cell carcinoma (SCC) [4, 8]. Previous studies have reported that circulating tumor cell (CTC) clusters trigger breast cancer metastasis more strongly than non-clustered CTC [9, 10], and an increased number of CTC clusters in the blood stream is associated with a worse prognosis in breast cancer patients [10, 11]. Therefore, collective invasion is considered a critical mode of cancer cell invasion for determining the prognosis of patients.

Recently, it has been reported that collective invasion occurs in a cancer cell cluster consisting of polyclonal cancer cells [12–14]. This leads to metastatic foci consisting of polyclonal cancer cells, causing heterogeneity of metastases [9, 12–15]. In targeted therapy, minority cancer cells that are not targeted by drugs can survive [16]. In addition, minority cancer cells support drug resistance and growth in dominant cancer cells [17]. Thus, the heterogeneity of cancer cells is associated with difficulties in targeted cancer therapy. Since collective invasion generates heterogeneity of metastases, inhibition of collective invasion is considered to improve cancer treatment. Attacking the cells that drive collective invasion in polyclonal cancer cells could achieve this inhibition; however, such cells are not well understood. Thus, we carried out subcloning of a cancer cell population to identify the cells responsible for collective invasion, and upregulated signaling pathways in these cells were investigated.

Signal transducer and activator of transcription 1 (STAT1) is a transcription factor that responds to interferons [18, 19] and is universally expressed in a variety of cell types, including cancer cells [20]. STAT1 induces apoptosis by enhancing the expression of caspase 1 in human carcinoma cells and lymphoma cells, as well as in spontaneously developed lens cancers by enhancing ectopic expression of the SV40 oncogene in mice [21–23]. In contrast, it has been reported that high STAT1 expression enhanced proliferation, invasiveness, and resistance to apoptosis in colorectal and esophageal carcinoma in vivo and in vitro [24, 25]. Therefore, the impact of STAT1 on cancer is not yet fully understood because the role of STAT1 varies among cancer cell types and situations. In addition, its role in collective invasion remains unclear.

Intercellular adhesion is an important factor that determines the characteristics of cancer cell clusters. For instance, the expression of genes encoding cell–cell adhesion molecules, such as E-cadherin and p120-catenin, is required for the collective invasion of SCC [26–28]. Although some studies have shown the relationship between the expression of genes involved in cell–cell adhesion and the behavior of cancer cell clusters, the detailed intercellular structure remains largely unknown. A recent study proposed that the localization of soluble factors in sealed intercellular spaces of cancer cell clusters promotes malignancy in triple-negative breast cancer without the expression of receptors for estrogen, progesterone, and human epidermal growth factor [29]. This study was conducted ultrastructural analysis of intercellular structures using electron microscope, and demonstrated the importance of intercellular structures in addition to changes in gene expression and signal transduction.

In this study, we established subclones with different invasive potential from a skin SCC line, A431 cells, which consist of heterogeneous cells and can invade as a polyclonal cell cluster [30]. We also found that A431 subclone with high-invasive potential has elevated STAT1 activity. In addition, cell clusters of the high-invasive A431 subclone have a hermetically sealed and narrow intercellular structure where interferon-β (IFNB) is localized, promoting collective invasion via STAT1 activation. The mixed spheroids of the high- and low-invasive subclones showed that the high-invasive STAT1-activated sub-clonal cells were located at the invasive front of the invasive protrusion and led to collective invasion with the low-invasive sub-clonal cells. Furthermore, immunohistochemical analysis of STAT1 protein in the tissue microarray of skin SCC revealed that STAT1 is notably expressed in the invasive front of cancer cell clusters during collective invasion. These findings indicate that the IFNB/STAT1 axis promotes the collective invasion of cancer cells with sealed intercellular structure and that cancer cells with elevated STAT1 signaling drive the collective invasion of SCC.

## Results

### Establishment of subclones with high- or low-invasive potentials from A431 cells consisted of heterogeneous cells

**Fig. 1:**
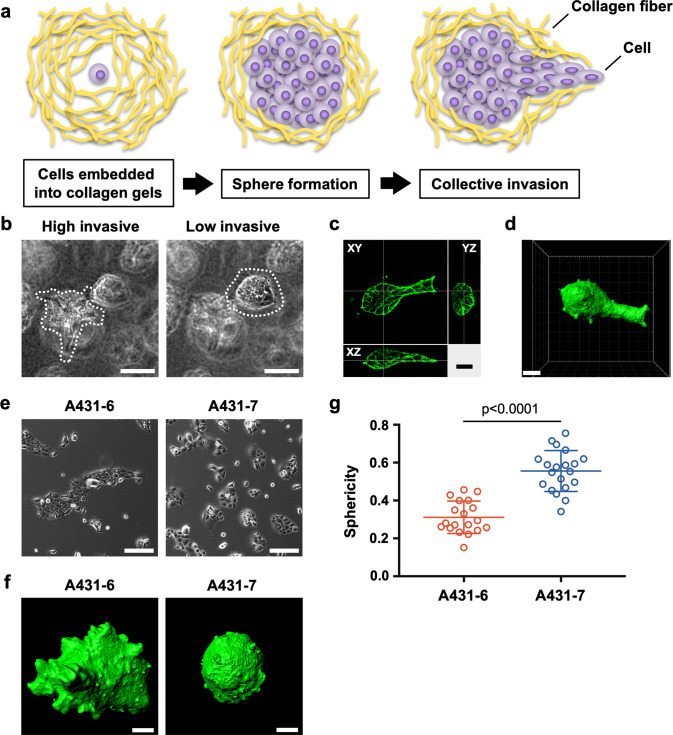
Establishment of subclones with high- or low-invasive potentials from A431 cells consisted of heterogeneous cells. **a** Schematic illustration of the experimental system to assess collective invasion. **b** Representative phase-contrast images of the invasive cell cluster (left) and non-invasive cell cluster (right) in A431-WT cells cultured in a three-dimensional collagen gel culture system; both were captured in the same field. The dotted lines show the contours of the cell clusters. Scale bars represent 100 µm. **c** F-actin staining of the invasive cell cluster in A431-WT cells cultured in three-dimensional collagen gel culture with XY, YZ, and XZ sectional views. Scale bar represents 25 µm. **d** Three-dimensional constructed image of (**c**) using the Imaris software. Scale bar represents 30 µm. **e** Phase contrast images of the high-invasive A431-6 and low-invasive A431-7 subclones. Scale bars represent 200 µm. **f** Representative three-dimensional constructed images of A431-6 and A431-7 cell clusters cultured in a three-dimensional collagen gel culture system. The sphericity of A431-6: 0.221 and A431-7: 0.715. Scale bars represent 30 µm. **g** Quantification of sphericity in (**f**). Lines show the mean with standard deviation (SD) for *n* = 19 (A431-6) and *n* = 19 (A431-7) clusters by two independent experiments.

Collective cancer cell invasion was evaluated using a spheroid invasion assay in a collagen gel (Fig. 1a). To assess the invasive potential of each cell in a cancer cell line, cells were embedded in collagen gels at a low cell density (6 × 10^4^ cells in 600 µL of collagen gel). In this experimental system, single cell-derived spheroids were formed by seeding cells at a low density, and the spheroids invaded the surrounding extracellular matrix. This invasive property of cells was used to assess the invasiveness of cancer cells. First, we examined the invasiveness of heterogeneous A431-wild type (WT) cells by culturing in this experimental system and found that A431-WT cells consisted of invasive and non-invasive cells (Fig. 1b); invasive cell clusters formed invasive protrusions into the surrounding matrix, whereas non-invasive cell clusters showed a round-shaped morphology without invasive protrusion, suggesting that A431-WT cells are a mixture of cells with various invasive potential. In addition, the invasive protrusion of invasive cells consisted of multiple cells, indicating that collective invasion occurred (Fig. 1c). The Imaris software was used for three-dimensional image construction in order to calculate the sphericity of cell clusters based on their surface area and volume to quantify collective invasion (Fig. 1d and Movie 1). When a cell cluster exhibits a round morphology without invasion, its sphericity value approaches 1 (Fig. S1a). Conversely, when a cell cluster invades the surrounding matrix, the surface area of the cell cluster increases because of the bumpy surface; therefore, its sphericity approaches zero (Fig. S1a). Thus, because sphericity and invasiveness are inversely correlated, we defined sphericity as an index of collective invasion. Since A431-WT cells are a mixture of heterogeneous cells, subcloning of A431-WT cells was carried out to establish subclones with different invasive potentials. We established a total of 20 subclones (A431-1 to A431-20) and examined their sphericity as an indicator for invasiveness (Fig. S1b). A431-3, 8, 12, 18, and 19 were omitted because they did not form a cell cluster (e.g., apoptosis or no cell–cell junction) that could be evaluated. From the data, we selected A431-6 as a high-invasive subclone and A431-7 as a low-invasive subclone, which displayed bumpy-shaped morphology with invasion and round-shaped morphology without invasion, respectively (Fig. 1e, f). We confirmed that the sphericity of A431-6 cell clusters was observed to be significantly lower than that of A431-7 cell clusters (Fig. 1g). Furthermore, the volume of A431-6 cell clusters was significantly larger than that of A431-7 cell clusters, suggesting that the proliferative capacity of the A431-6 subclone was higher than that of A431-7 (Fig. S1c). In addition, the A431-6 and A431-7 subclones retained their differences in invasiveness, even after undergoing 13 passages each (Fig. S1d). Taken together, we succeeded in establishing subclones with different invasiveness and a novel and unique system for evaluating collective invasion by analyzing the sphericity of cell clusters.

### STAT1 plays a crucial role in collective invasion of high-invasive A431-6 cells

**Fig. 2:**
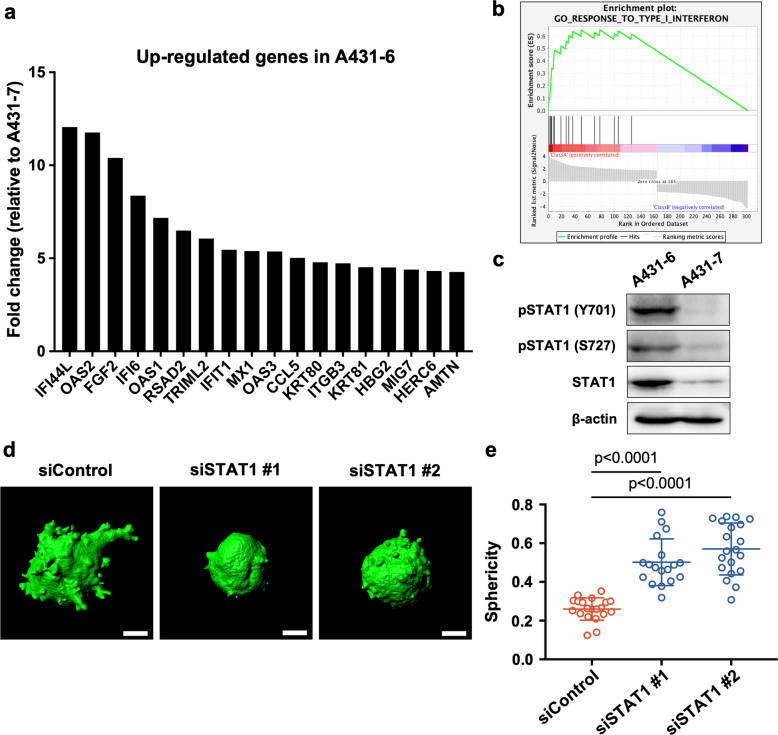
STAT1 plays a crucial role in collective invasion of high-invasive A431-6 cells. **a** The DNA microarray result comparing the A431-6 and A431-7 subclones. Genes with more than a 4-fold increase in A431-6 are shown. **b** GSEA showing increased response to type-I interferon in A431-6 cells. **c** Representative western blotting images for pSTAT1 (Y701), pSTAT1 (S727), STAT1, and β-actin in A431-6 and A431-7 cells. This experiment was repeated three times, and similar results were obtained in all experiments. **d** Representative three-dimensional constructed images of siControl-, siSTAT1 #1- or STAT1 #2-treated A431-6 cell clusters cultured in a three-dimensional collagen gel culture system. The sphericity of siControl: 0.263, siSTAT1#1: 0.638, and siSTAT1#2: 0.633. Scale bars represent 30 µm. **e** Quantification of sphericity in (**d**). Lines show the mean with SD for *n* = 20 (siControl), *n* = 18 (siSTAT1#1), and *n* = 20 (siSTAT1#2) clusters by two independent experiments.

Although A431-6 and A431-7 cells were derived from the same parental cell line, their invasive abilities were significantly different. Thus, we hypothesized that comparing the gene expression profiles of A431-6 and A431-7 cells would be effective for revealing the molecular mechanisms underlying the collective invasion of the cells. We then carried out a DNA microarray of GeneChip to identify the genes that are differentially expressed in A431-6 and A431-7 cells. DNA microarray analysis revealed that several genes induced by type I interferon, such as interferon-induced protein 44 like and 2’-5’-oligoadenylate synthetase 2, were markedly upregulated in A431-6 cells compared to those in A431-7 cells (Fig. 2a and Supplementary Dataset). In addition, we confirmed the reproducibility of the microarray results using quantitative PCR (qPCR) (Fig. S2a). Gene set enrichment analysis (GSEA) showed the enrichment of response to type-I interferon in A431-6 cells (Fig. 2b). These data strongly suggest that the enhancement of the type-I interferon pathway directs collective invasion.

Type-I interferon activates the Janus kinase (JAK)-STAT1 pathway by interacting with the type-I interferon receptor (IFNAR). STAT1 is phosphorylated by JAK and translocated to the nucleus, where it acts as a transcription factor [31, 32]. Since many genes highly expressed in A431-6 cells have been reported to be transcribed by STAT1 [33], we performed western blotting to compare the protein levels of total and phosphorylated STAT1 in each subclone. In A431-7 cells, both STAT1 and phosphorylated STAT1 were detected at negligible levels, whereas A431-6 cells showed notable high expression levels (Figs. 2c and S2b), suggesting that the differential activity of STAT1 generates differential gene expression between A431-6 and A431-7 cells. Furthermore, the depletion of STAT1 by siRNAs increased the sphericity of the cell clusters (Figs. 2d, e and S2c), which suppressed collective invasion. In addition, STAT1 depletion did not affect the volume of the cell clusters, suggesting that STAT1 controls collective invasion without affecting cell proliferation (Fig. S2d). Thus, factors other than STAT1 might have caused a difference in the proliferative capacity between A431-6 and A431-7 cells. We also confirmed that STAT1 depletion suppressed the collective invasion of the parental A431-WT cells (Fig. S2e). These findings indicate that STAT1 plays a crucial role in the collective invasion of high-invasive A431-6 and A431-WT cells.

### JAK and IFNAR are upstream molecules responsible for phosphorylation of STAT1, contributing collective invasive potential

**Fig. 3:**
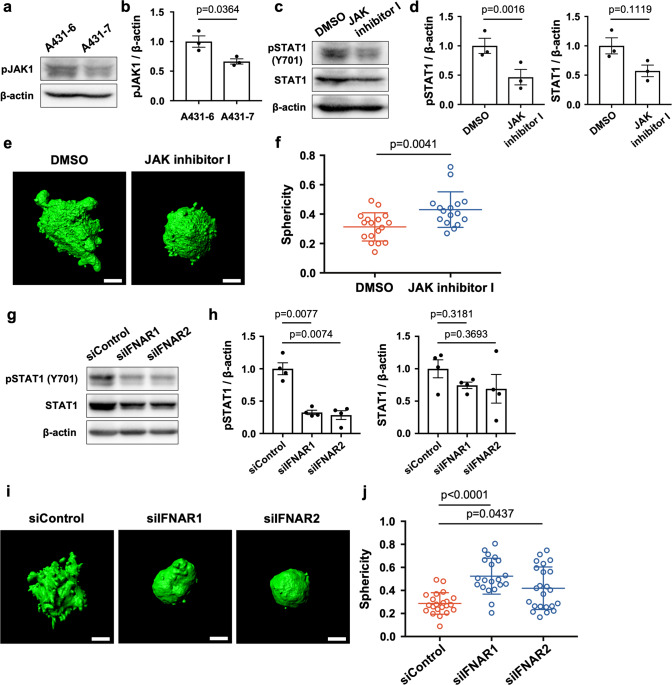
JAK and IFNAR are upstream molecules responsible for phosphorylation of STAT1, increasing collective invasive potential. **a** Representative western blotting images for pJAK1 and β-actin in A431-6 and A431-7 cells. **b** Quantification of (**a**). Bars represent the mean ± standard error of the mean (SEM) of three independent experiments. **c** Representative western blotting images for pSTAT1 (Y701), STAT1, and β-actin in DMSO or JAK inhibitor I (15 nM)-treated A431-6 cells. **d** Quantification of (**c**). Bars represent the mean ± standard error of the mean (SEM) of three independent experiments. **e** Representative three-dimensional constructed images of DMSO or JAK inhibitor I (15 nM)-treated A431-6 cell clusters cultured in a three-dimensional collagen gel culture system. The sphericity of DMSO: 0.247, JAK inhibitor I: 0.671. Scale bars represent 30 µm. **f** Quantification of sphericity in (**d**). Lines show the mean with SD for *n* = 17 (DMSO) and *n* = 16 (JAK inhibitor I) clusters by two independent experiments. **g** Representative western blotting images for pSTAT1 (Y701), STAT1, and β-actin in siControl-, siIFNAR1-, or siIFNAR2-treated A431-6 cells. **h** Quantification of protein bands in (**f**). Bars represent the mean ± SEM of four independent experiments. **i** Representative three-dimensional constructed images of siControl, siIFNAR1, or siIFNAR2-treated A431-6 cell clusters cultured a in three-dimensional collagen gel culture system. The sphericity of siControl: 0.176, siIFNAR1: 0.688, and siIFNAR2: 0.712. Scale bars represent 30 µm. **j** Quantification of sphericity in (**h**). Lines show the mean with SD for *n* = 22 (siControl), *n* = 20 (siIFNAR1), and *n* = 23 (siIFNAR2) clusters by two independent experiments.

In the type-I interferon pathway, JAK1 is a representative kinase that phosphorylates STAT1 [31]. Because there is a notable difference in the phosphorylation of STAT1 between A431-6 and A431-7 cells, western blotting of phosphorylated JAK1 was carried out in each subclone. As expected, phosphorylated JAK1 in A431-6 cells was significantly higher than that in A431-7 cells (Fig. 3a, b). A431-6 cells were treated with JAK inhibitor I, an inhibitor of all JAKs (JAK1, JAK2, JAK3, and TYK2, at IC50 (15 nM)) to investigate the contribution of JAKs to phosphorylation of STAT1. Since JAK inhibitor I significantly depleted phosphorylated STAT1 in A431-6 cells (Fig. 3c, d), it is conceivable that STAT1 is phosphorylated in a JAK-dependent manner. Subsequently, we examined the influence of JAK inhibition on the sphericity of the A431-6 cell clusters. JAK inhibitor I treatment in A431-6 cells significantly increased sphericity, that is, decreased the collective invasion potential (Fig. 3e, f). These data demonstrate that JAK plays an important role in the collective invasion of A431-6 cell clusters by modulating STAT1 activity.

IFNAR, a representative type-I interferon receptor, is a heterodimer consisting of IFNAR1 and IFNAR2 [32]. JAK is coupled to IFNARs and transmits signals from the ligand of IFNAR to STAT1 [31, 32]. Next, we examined whether IFNAR depletion suppressed the phosphorylation of STAT1 and collective invasion in A431-6 cells. The depletion of IFNAR1 and IFNAR2 by siRNA significantly decreased the amount of phosphorylated STAT1 (Figs. 3g, h and S3). Moreover, the sphericity of A431-6 cell clusters was notably increased by siRNA treatment (Fig. 3i, j). These findings demonstrate that the expression of IFNAR1 and IFNAR2 is required for the phosphorylation of STAT1 and collective invasion in A431-6 cell clusters. These data suggest both JAK and IFNAR contribute to collective invasion by modulating STAT1activity.

### Interferon-β promotes the collective invasion of cancer cells with sealed intercellular spaces

**Fig. 4:**
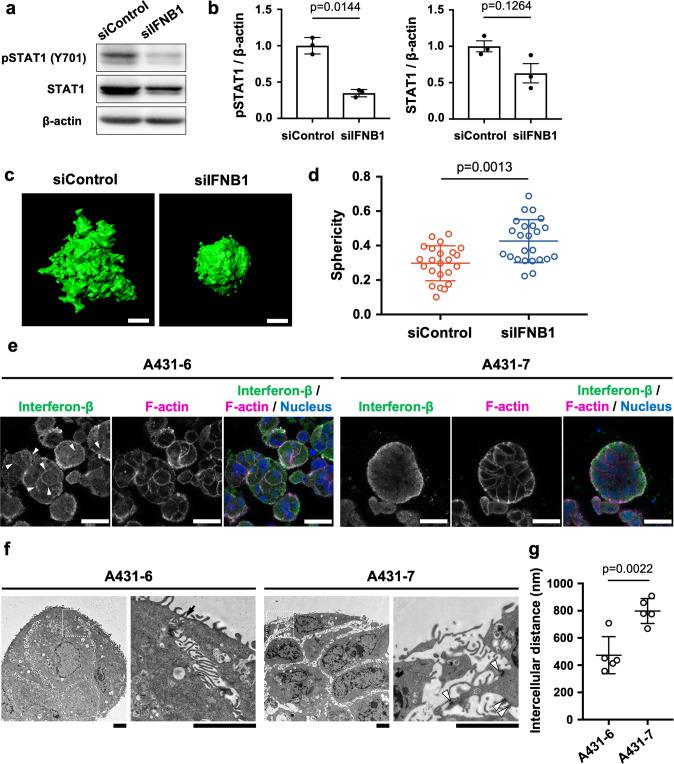
Interferon-β promotes the collective invasion of cancer cells with sealed intercellular spaces. **a** Representative western blotting images for pSTAT1 (Y701), STAT1, and β-actin in siControl- or siIFNB1 -treated A431-6 cells. **b** Quantification of protein bands in (**a**). Bars represent the mean ± SEM of three independent experiments. **c** Representative three-dimensional constructed images of siControl- or siIFNB1-treated A431-6 cell clusters cultured in a three-dimensional collagen gel culture system. The sphericity of siControl: 0.147 and siIFNB1: 0.558. Scale bars represent 30 µm. **d** Quantification of sphericity in (**c**). Lines show the mean with SD for *n* = 23 (siControl) and *n* = 24 (siIFNB1) clusters by two independent experiments. **e** Immunofluorescence images for interferon-β (INFB; green) with F-actin (magenta) and nucleus (blue) in A431-6 and A431-7 cell clusters in a three-dimensional collagen gel culture system. Arrowheads show immunoreactivity for IFNB in the intercellular spaces. Scale bars represent 25 µm. **f** Transmission electron microscopy of A431-6 and A431-7 cell clusters cultured in a three-dimensional collagen gel culture system. The regions surrounded by the white dotted squares are magnified on the right. The arrow shows the sealed edge of the intercellular space. Arrowheads show intercellular junctions. Scale bars represent 3 µm. **g** Quantification of intercellular distances in (**f**). Lines show the mean with SD in five clusters.

Type-I interferons include interferon-α (IFNA) and interferon-β (IFNB), and both can bind to IFNAR [32]. To investigate the function of IFNA and IFNB in A431-6 cells, IFN alpha-IFNAR-IN-1, a low-molecular-weight inhibitor of the IFNA and IFNAR interaction, was added to A431-6 cells. IFN alpha-IFNAR-IN-1 treatment did not affect the amount of phosphorylated STAT1 in A431-6 cells (Fig. S4). Subsequently, knockdown of IFNB1 was generated by siRNA in A431-6 cells to assess the relationship between STAT1 and IFNB1. Phosphorylated STAT1 was significantly decreased (Figs. 4a, b and S3) and the sphericity was increased; that is, collective invasion was decreased by IFNB1 depletion (Fig. 4c, d). These data indicate that IFNB, but not IFNA, promotes collective invasion through the activation of STAT1 in A431-6 cell clusters.

To assess how IFNB contributes to STAT1 activity and collective invasion, we performed immunofluorescence staining for IFNB. IFNB immunoreactivity was localized to the intercellular sites in A431-6 cell clusters (arrowheads in Fig. 4e), whereas there were no obvious immunoreactivities in the intercellular sites in A431-7 cell clusters (Fig. 4e). There was no significant difference between A431-6 and A431-7 cells in the intensity of IFNB immunoreactivity on the surface of cell clusters. Some colonies of A431-WT cells also expressed IFNB in the intercellular site (Fig. S5). However, the mRNA levels of IFNB and its receptors were higher in A431-7 cells than in A431-6 cells (Fig. S6a). Enzyme-linked immunosorbent assay (ELISA) results also showed that the culture supernatant of A431-7 cells had a higher IFNB concentration than that of A431-6 cells (Fig. S6b). Furthermore, the addition of recombinant human IFNB did not affect the sphericity and collective invasion of A431-7 cell clusters (Fig. S6c). When IFNB neutralizing antibody was added to inhibit the activity of extracellular IFNB, there was no change in the invasive ability of A431-6 cell clusters (Fig. S6d). In addition, we confirmed that its receptor, IFNAR2, is expressed in the intercellular site of A431-6 cells (Fig. S6e). These data suggest that intercellular IFNB, but not extracellular IFNB, is responsible for collective invasion.

Recent research has shown that the sealed cell–cell adhesion structure promotes the accumulation of soluble factors such as epidermal growth factor receptor (EGFR) ligands at intercellular sites, whereby triple-negative breast cancer becomes malignant [29]. To investigate the ultrastructural difference, especially cell–cell adhesions, between A431-6 and A431-7 cell clusters, transmission electron microscopy (TEM) was performed. We found that the edges of A431-6 cell clusters were sealed, whereas those of the A431-7 cell clusters were not sealed (Fig. 4f), although intercellular junctions were well-developed in A431-7 cell clusters but not in A431-6 cell clusters. Furthermore, the intercellular spaces of the A431-7 cell clusters were remarkably wider than those of the A431-6 cell clusters (Fig. 4g). Next, immunoelectron microscopy was carried out to investigate the localization of intercellular IFNB in detail. As a result, immunolabeling of IFNBs adhering to the cell surface in the intercellular spaces of A431-6 cells was performed (Fig. S7a). The cells were then treated with ethylene glycol tetraacetic acid (EGTA), a calcium-selective chelator, to open the sealed intercellular space. 5 mM of EGTA disrupted intercellular adhesion, whereas 1 mM loosened intercellular adhesion. In both cases, intercellular IFNB was not detected by immunofluorescent staining (Fig. S7b). Since cells retained a cell cluster morphology in 1 mM EGTA, sphericity was measured and was significantly higher than that of non-treated cells (Fig. S7c). It is possible that the sealed edge of the intercellular spaces prevents secreted INFB from leaking out of A431-6 cell clusters. Collectively, these data suggest that the sealed and narrow intercellular spaces in A431-6 cell clusters contribute to the IFNB-induced increase in phosphorylation of STAT1 and elevated collective invasiveness.

### Depletion of keratin (KRT) leads the formation of sealed and narrow intercellular structure and STAT1 activation

Next, we focused on the upregulated genes in A431-7 cells compared to those in A431-6 cells, which may be related to their low-invasive potential. Microarray results showed that keratin genes, such as KRT1, KRT10, and KRT14 were notably upregulated in A431-7 cells (Fig. S8a). GSEA also showed the enrichment of keratinization in A431-7 cells (Fig. S8b). We confirmed the reproducibility of the microarray results using qPCR (Fig. S8c). Keratin filament, a typical cytoskeletal component in squamous epithelial cells, is involved in the formation of intercellular adhesions such as desmosomes [34, 35]. We assumed that upregulated keratin genes in A431-7 cells influenced the morphology of the intercellular structure; thus, keratin gene knockdown experiments using siRNAs were performed (Fig. S9a). Interestingly, KRT1-knockdown A431-7 cell clusters possessed narrow intercellular spaces, and the edges of the cell cluster were sealed like those of A431-6 cell clusters (Fig. S9b and Fig. 4f). Furthermore, the quantitative analysis demonstrated that the intercellular spaces of KRT1-knockdown A431-7 cell clusters were significantly narrower than those of control A431-7 cell clusters (Fig. S9c). Moreover, the phosphorylation of STAT1 and the total amount of STAT1 proteins were significantly increased by the depletion of keratin genes, especially KRT1 and KRT10 (Figs. S9d and S9e). In summary, we clarified that keratin genes are involved in the formation of wider and non-sealed intercellular spaces of A431-7 cell clusters, and the depletion of keratin genes promotes sealed intercellular structure generation and subsequent STAT1 activation.

### Direct contact with high-invasive subclone drives invasion of low-invasive subclone

**Fig. 5:**
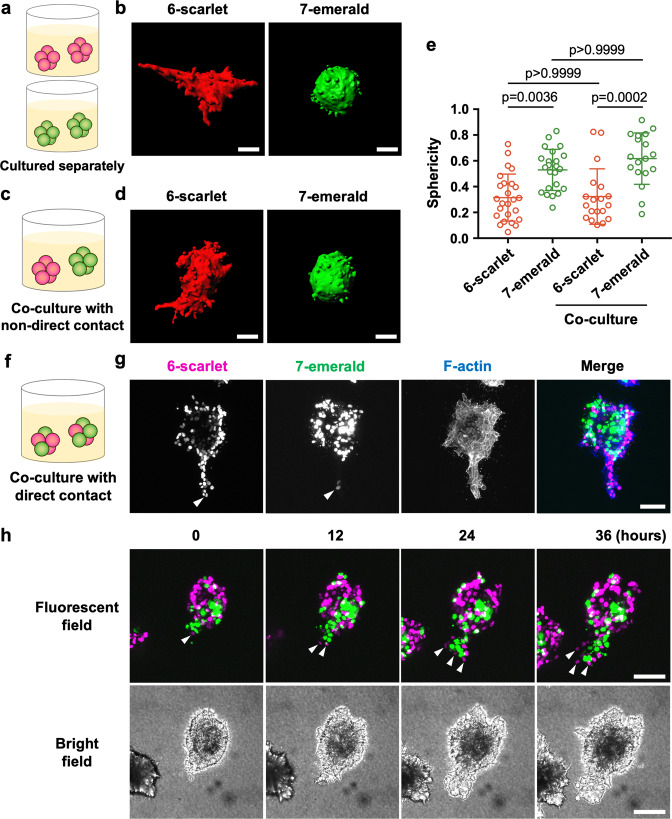
Direct contact with high-invasive subclone leads invasion of low-invasive subclone. **a** Schematic illustration of the culture of A431-6 cells expressing scarlet-histone H2B (6-scarlet, red) and A431-7 cells expressing emerald-histone H2B (7-emerald, green) cell clusters. **b** Representative three-dimensional constructed images of (**a**). 6-scarlet and 7-emerald cell clusters were cultured in a three-dimensional collagen gel culture system. The sphericity of 6-scarlet: 0.099 and 7-emerald: 0.596. Scale bars represent 30 µm. **c** Schematic illustration of the experiment culturing 6-scarlet and 7-emerald cell clusters with non-direct contact. **d** Representative three-dimensional constructed images of (**c**). 6-scarlet and 7-emerald cell clusters were cultured in a three-dimensional collagen gel culture system. The sphericity of 6-scarlet: 0.137 and 7-emerald: 0.604. Scale bars represent 30 µm. **e** Quantification of sphericity in (**b**, **d**). Lines show the mean with SD in >16 clusters by two independent experiments. **f** Schematic illustration of the experiment with mixed spheroids of 6-scarlet and 7-emerald cells in direct contact. **g** Representative images of a mixed spheroid consisting of 6-scarlet (magenta) and 7-emerald (green) cells cultured in a collagen gel. Arrowheads show invading sub-clonal cells. The merged image is shown by F-actin (blue). Scale bars represent 30 µm. **h** Time-lapse imaging of a mixed spheroid consisting of 6-scarlet (magenta) and 7-emerald (green) cells cultured in a collagen gel. Arrowheads show 6-scarlet cells leading to polyclonal collective invasion. Scale bars represent 100 µm.

Several studies have reported that cancer cells with high-invasive potential drive the invasion of low-invasive cancer cells through both direct and non-direct interactions [15, 36, 37]. Because an investigation on the interaction between phenotypically different cells is important for understanding collective invasion by polyclonal cancer cells, co-culture experiments with high-invasive A431-6 and low-invasive A431-7 sub-clonal cells were carried out. First, we developed A431-6-scarlet-histone H2B (6-scarlet) and A431-7-emerald-histone-H2B (7-emerald) cells to distinguish the subclones in the co-culture environment. There was no significant change in invasive potential by histone modification: 6-scarlet cell clusters with low sphericity scores invaded the surrounding matrix, while 7-emerald cell clusters with high sphericity scores did not (Fig. 5a, b, and e). Subsequently, we investigated whether non-direct or direct interaction of high-invasive 6-scarlet cells and low-invasive 7-emerald cells influences the collective invasion potential. To assess the effect of non-direct interaction, both 6-scarlet and 7-emerald cells were seeded together in a collagen gel, where each subclone formed independent cell clusters (Fig. 5c). There was no significant influence of the non-direct contact co-cultures (Fig. 5d, e): 6-scarlet cell clusters showed high invasive potential, whereas the invasiveness of 7-emerald cell clusters did not change. Next, we performed co-culture with direct contact to assess the effects of cell–cell contact between the subclones with different invasiveness (Fig. 5f). To achieve this, mixed spheroids of 6-scarlet and 7-emerald cells were prepared using the hanging drop method and cultured in a collagen gel until collective invasion was observed. Intriguingly, 7-emerald cells were included in the collective invasion chain consisting of 6-scarlet cells, and polyclonal collective invasion was occurred (Fig. 5g). In addition, time-lapse imaging showed that 6-scarlet cells were present at the invasive front of the collective invasion, and 7-emerald cells appeared to follow it (Fig. 5h and Movie 2). Furthermore, immunofluorescence staining revealed that the nuclear localization of STAT1 was notably enhanced in the cells, leading to polyclonal collective invasion of 6-scarlet and 7-emerald (Fig. S10). We then prepared mixed spheroids of IFNB-knockdown and non-treated A431-6 cells, and examined whether the collective invasion chain contained IFNB-knockdown cells. To distinguish knockdown cells from non-treated cells, 6-scarlet cells were transfected with IFNB siRNA and mixed with A431-6 cells without the fluorescent tag. As a result, the collective invasion chain mainly contained non-treated cells surrounding INFB knockdown cells (Fig. S11). These data suggest that direct interaction of the low-invasive sub-clonal cells and high-invasive sub-clonal cells drives polyclonal collective invasion, where high-invasive sub-clonal cells lead to low-invasive sub-clonal cells.

### STAT1 is highly expressed at the invasive front of human skin SCCs

**Fig. 6:**
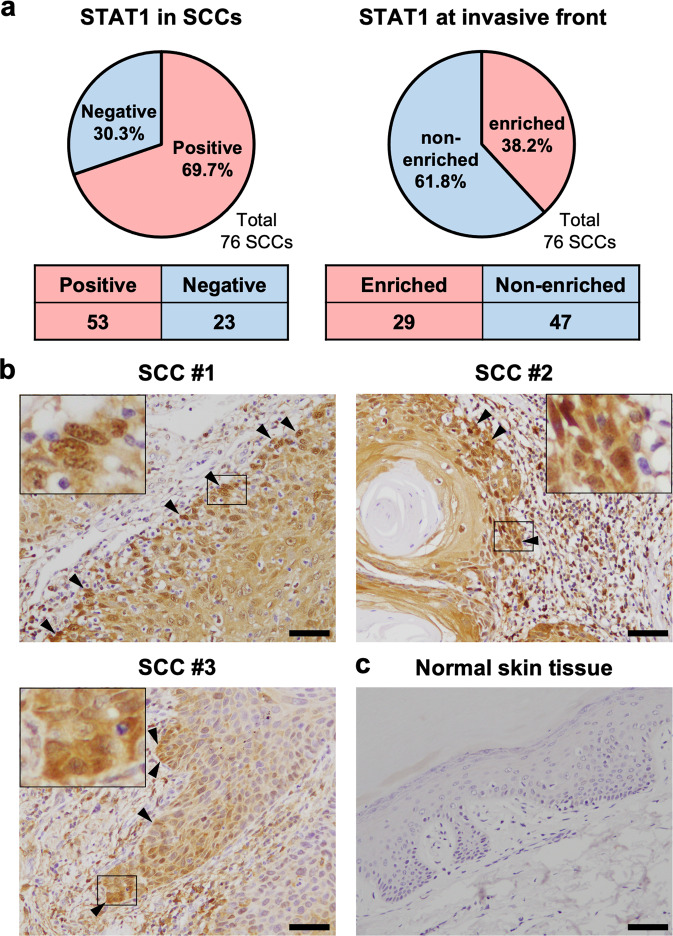
STAT1 is highly expressed at the invasive front of skin SCCs. **a** The pie charts and tables show the percentage and number of STAT1 positive skin squamous cell carcinoma (SCC) in human tissue microarray (left) and those of SCC with enriched STAT1 at the invasive front (right), respectively. The results were independently assessed by two pathologists. **b** Representative images showing enrichment of STAT1 in the invasive front of SCC. Arrowheads indicate high STAT1 expression in leading cancer cells at the invasive fronts of SCC cancer cell groups. The regions surrounded by squares were magnified. The scale bars represent 50 µm. **c** Representative images showing STAT1-negative in normal skin tissues. Scale bars represent 50 µm.

Finally, immunohistochemistry for STAT1 was performed on 76 samples of skin SCC patients and four samples of normal skin tissue in the human tissue microarray of skin SCCs. The results were independently assessed by two pathologists. Immunohistochemistry showed that 53 of 76 SCCs (69.7%) were STAT1-positive (Fig. 6a). In addition, the enrichment of STAT1 at the invasive front was observed in 29 of 76 SCCs (38.2%) (Fig. 6a, b). As shown in Fig. 6b, STAT1 was highly expressed and significantly localized to the nucleus in SCC cells adjacent to the stroma, specifically, cells at the invasive front. At this time, STAT1 staining was negative in all normal tissues (Fig. 6c). Furthermore, enrichment of STAT1 expression in the nuclei of cancer cells located at the edges of cancer cell nests was also observed in some cases of human SCC in situ (Bowen’s disease), supporting the notion that STAT1 expression may be a prerequisite for initiating or promoting the collective invasion of cancer cells (Fig. S12). Taken together, these data suggest that STAT1-high expressing cells drive polyclonal collective invasion by leading STAT1-low expressing cells in SCC patients.

## Discussion

This study demonstrates that the interferon-β/STAT1 axis drives the collective invasion of cancer cells with sealed intercellular spaces, and that STAT1-activated cells drive polyclonal collective invasion through low-invasive sub-clonal cells. A schematic illustration of the results of the present study is shown in Fig. S13. We found that the high-invasive cell clusters have a hermitically sealed and narrow intercellular structure, while low-invasive cell clusters have a non-sealed and wide intercellular structure. INFB is localized to the sealed intercellular spaces of high-invasive cell clusters and promotes phosphorylation of STAT1 and collective invasion through the activation of the IFNAR and JAK pathways. Co-culture experiments with a mixed spheroid of subclones, where high-invasive cells and low-invasive cells directly contact each other, showed that high-invasive sub-clonal cells drive polyclonal collective invasion by leading low-invasive sub-clonal cells. Furthermore, immunohistochemical analysis of tissue microarray revealed the enrichment of STAT1 at the invasive front of human skin SCCs.

We succeeded in obtaining subclones with high- or low-invasive potentials from A431 SCC cells consisting of polyclonal cells with heterogeneous invasiveness and established a novel unique evaluation system based on the morphology of whole cell clusters. In conventional experimental systems, it is difficult to visualize whole cell clusters by embedding artificially prepared spheroids into the extracellular matrix because of the size of the spheroid. In contrast, using this experimental system, we can assess the behavior of <100 cell clusters; therefore, we were able to evaluate the morphology of the whole cell cluster. By analyzing the sphericity of the whole cell cluster, we could evaluate the invasive potential of each cell cluster. However, there is a problem with this experimental system: the sphericity varies even without invasion. For instance, even if the surface of the cell cluster is very bumpy without invasion, the sphericity decreases. Therefore, since sphericity acts only as an index, it is necessary to determine whether invasion actually occurs from the imaging data.

By comparing gene expression profiles between subclones with high- and low-invasive potentials through DNA microarray, we determined that the activity of STAT1 drives collective invasion. Previous studies have reported that STAT1 contributes to migration and invasion in a variety of cancer cell types [24, 25, 38, 39]. One study showed that STAT1 modulates the binding of cells to fibronectin by forming a complex with focal adhesion kinase (FAK) in focal adhesion; moreover, it enhances cell migration as determined by Transwell migration assay [39]. Previously, we showed that integrin-β1, a major regulator of FAK, plays a crucial role in the two-dimensional collective invasion of parental A431 cells [30]. These findings suggest that STAT1 contributes to collective invasion by modulating the functions of integrin-β1 and FAK.

We found that cell clusters of high-invasive sub-clonal cells have sealed and narrow intercellular spaces where INFB is localized. A recent study reported that the accumulation of soluble factors, such as EGFR ligand, in the sealed intercellular space defined as “nanolumina” contributes to the malignancy of triple-negative breast cancer [29]. The clusters of high-invasive subclone have hermetically sealed intercellular structures that resemble “nanolumina”, and localized INFB promotes collective invasion via STAT1 activation. Furthermore, the present study identified KRT1 as one of the factors that negatively regulates the formation of “nanolumina”-like structure. Intriguingly, well-developed intercellular junctions were found in the low-invasive subclone with non-sealed and wider intercellular spaces, rather than in the high-invasive subclone. DNA microarray analysis also showed that the expression of genes involved in intercellular junctions (e.g., DSG1, desmoglein 1, GJA1, gap junction protein alpha 1) was higher in A431-7 than in A431-6 (Supplementary Dataset). These results suggest that the sealed intercellular structures and the width of intercellular spaces in cancer cell clusters are independent of intercellular junctions. In normal keratinized squamous epithelial tissue of the skin, there are wide spaces between the cells, which are connected by intercellular bridges [40]. Even in well-differentiated SCCs with high keratin expression, intercellular bridges and wide intercellular spaces exist between cells [41]. The A431-7 subclone, with wide and unsealed intercellular spaces, had higher keratin expression than the A431-6 subclone, with narrow and sealed intercellular spaces. It is possible that a decrease in keratin expression, that is, the transition from a highly differentiated to a poorly differentiated form, results in narrower and sealed intercellular spaces.

An intercellular structure similar to “nanolumina” has been observed not only in the collective invasion of cancer cells, but also in collective migration during epithelial morphogenesis [42]. Thus, “nanolumina”-like structures are considered a universal phenomenon in diverse cell types; however, its biological significance has yet to be fully evaluated. This study demonstrated the significance of the sealed intercellular structure like “nanolumina” in collective invasion of cancer cells and is expected to accelerate future research about sealed intercellular structures.

We determined that the high-invasive subclone with high STAT1 activity was located at the invasive front of the invasive protrusion, resulting in collective invasion with the low-invasive subclone. Leader cells are located at the invasive front of the cell population and promote polyclonal collective invasion by leading low-invasive cells called follower cells [36, 37, 43, 44]. Our data indicate that cancer cells with high STAT1 activity act as leader cells during polyclonal collective invasion. In addition, previous studies have shown that enhanced fibronectin production in leader cells modulates the ability to drive follower cells in polyclonal collective invasion [37, 44]. In the present study, DNA microarray analysis revealed that the amount of fibronectin-coding mRNA was increased approximately three-fold in the A431-6 high-invasive compared to that in the A431-7 low-invasive subclone (Supplementary Dataset). This supports the hypothesis that A431-6 cells with high STAT1 activity function as leader cells. Furthermore, the tissue microarray of skin SCC samples revealed that STAT1 is notably expressed in the invasive front of SCCs, suggesting that STAT1 also works in leader cells in vivo to drive polyclonal collective invasion. However, the percentage of STAT1 enrichment cells at the invasive front of skin SCC was 38.2% (29/76), and there were STAT1-negative SCCs and STAT1-positive SCCs without enrichment of STAT1 at the invasive front. Integrin-β1, previously mentioned in relation to STAT1, has also been reported to be concentrated at the invasive front in skin SCC, and the percentage of integrin-β1 enrichment at the invasive front was 65% (8/13) [43]. Namely, it has been suggested that the enrichment of STAT1 at the invasive front is not remarkably higher than that of representative molecules such as integrin-β1. Thus, further investigations are needed to clarify whether STAT1 could be a therapeutic target for skin SCC.

The findings of this study demonstrate that the INFB/STAT1 axis promotes the collective invasion of cancer cells with sealed intercellular spaces. Furthermore, it has been suggested that cancer cells with STAT1 activation act as leader cells to drive the polyclonal collective invasion of skin SCC.

## Materials and methods

### A431 cell culture and the establishment of subclones with differential invasive potential

The human SCC cell line, A431 cells (American Type Culture Collection, Manassas, VA), were cultured in Dulbecco’s modified Eagle’s medium (Sigma-Aldrich Co. LLC, St Louis, MO, USA) supplemented with 10% fetal bovine serum (Sigma-Aldrich Co. LLC) and 1% antibiotic/antimycotic solution (Sigma-Aldrich Co. LLC). The cells were cultured in a humidified incubator at 37 °C with 5% CO_2_. A431 subclones were established using the limited dilution method. Parental A431 (A431-WT) cells were seeded in 96-well cell culture plates (Corning Incorporated, Corning, NY, USA) at 0.5 cells/well. We grew the cells only in the wells with a single colony. The first established subclone was named A431-1, and the subsequent subclones were named in the order in which they were established (A431-1 to A431-20). Among them, A431-6 cells showed high invasive potential, while A431-7 cells displayed low invasive activity, and we found that these subclones had a notable difference in the activity of the type-I interferon pathway by analyzing gene expression using DNA microarray as described below. Since the type-I interferon pathway is mediated by viral infection [32] and herpes virus is known as a pathogen of skin cancer [45], we tested the herpes virus infection using PCR, and the result was negative. In addition, a previous study reported that A431 cells were not infected with human papillomavirus, a pathogen of vulvar cancer [46]. All cell lines and subclones were tested for mycoplasma contamination using a mycoplasma detection kit (VenorGeM Mycoplasma Detection Kit, Sigma-Aldrich Co. LLC) according to the manufacturer’s instructions and did not exhibit any contamination symptoms after initial testing. Thus, we confirmed that the high type-I interferon pathway in A431-6 cells was not caused through infection by microorganisms. In all experiments except Fig. S1d, subclones with seven or fewer passages were used.

### Development of transgenic cell lines

The scarlet-Histone H2B encoding vector and emerald-Histone H2B encoding vector were constructed as described previously [30]. These vectors were used to distinguish A431-6 (6-scarlet) and A431-7 (7-emerald) cells in mixed cultured experiments [30]. Selective cultures were performed using a culture media containing 2 mg/mL puromycin after transfection into the subclones using Xfect transfection reagent (Takara Bio Inc., Shiga, Japan).

### DNA microarray

DNA microarray (Human Gene 2.0 ST Array; Applied Biosystems, Foster City, CA, USA) was performed using RNA derived from the subclones cultured in three-dimensional collagen gel culture. The data were analyzed by GSEA [47].

### Immunohistochemistry for tissue microarray of skin SCC

Immunohistochemistry for tissue microarray of invasive skin SCC (SK802b; US Biomax, Inc., Rockville, USA) and pre-invasive skin SCC, which is also known as SCC in situ or Bowen’s disease, was performed as described in a previous study [48]. The data were independently assessed by two pathologists.

Other relevant materials and methods can be found in Supplementary Information.

## Supplementary information

Movie 1

Movie 2

Supplementary Information

Supplementary Dataset

## Data Availability

All relevant data supporting our findings are available within the article and its supplementary information files or from the authors upon reasonable request. Source data are provided in this study.
