# Supplementary material for: The interferon-β/STAT1 axis drives the collective invasion of skin squamous cell carcinoma with sealed intercellular spaces
Source: Oncogenesis. 2022 May 24;11(1):27. doi: 10.1038/s41389-022-00403-9 (PMC9126940; doi:10.1038/s41389-022-00403-9)
Supplement: Supplementary file 3 — Supplementary Information [file 41389_2022_403_MOESM3_ESM.docx]

**Supplementary Figures**

**
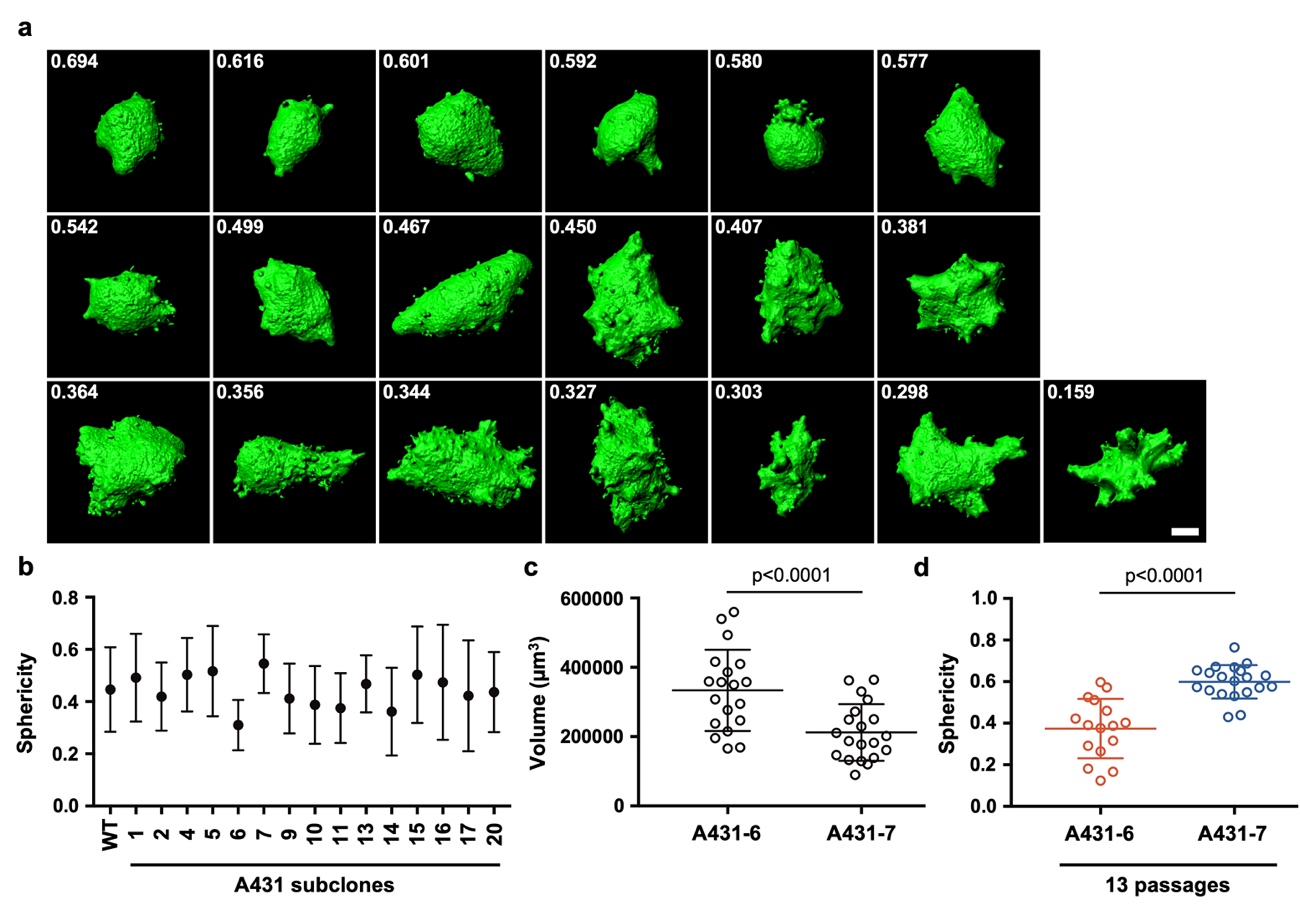
**

Supplementary Figure 1

1. Three-dimensional constructed images of A431-WT cell clusters cultured in a three-dimensional collagen gel culture system. The values show the sphericity of each cell cluster. Scale bars represent 30 µm.
2. Sphericity of the cell clusters in each subclone. The dots show the mean with standard deviation (SD) for 10 clusters.
3. Quantification of the cell cluster volume is shown in Fig. 1f. Lines show the mean with standard deviation (SD) for n=19 (A431-6) and n=20 (A431-7) clusters in two independent experiments.
4. Sphericity of cell clusters in the A431-6 and A431-7 subclones after 13 passages. Lines show the mean with standard deviation (SD) for n=16 (A431-6) and n=20 clusters in two independent experiments.


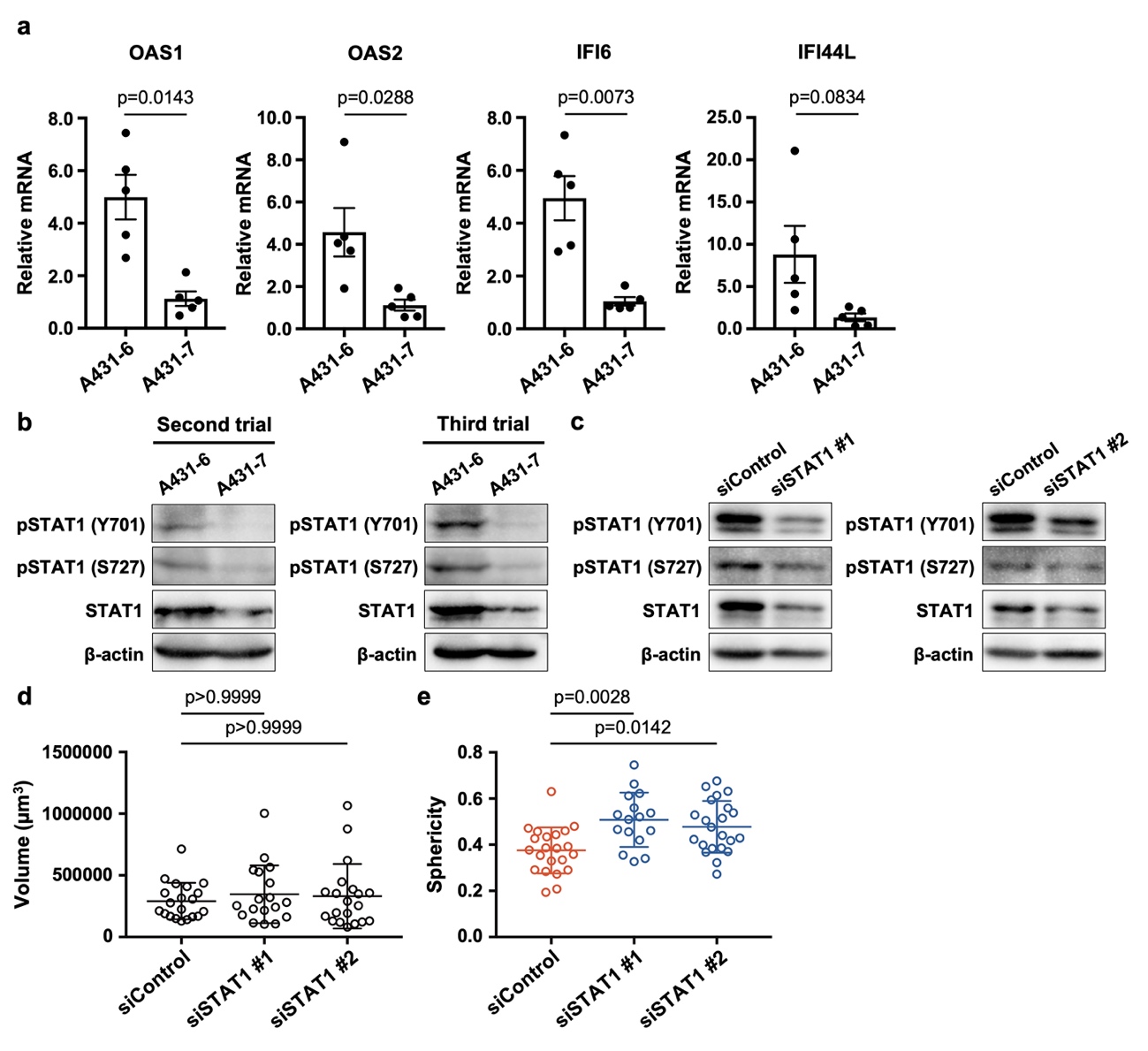
Supplementary Figure 2

1. Quantitative PCR (qPCR) results of the type-I interferon pathway-related genes (OAS; 2'-5'-oligoadenylate synthetase, IFI6; interferon alpha inducible protein 6, IFI44L; interferon-induced protein 44 like) in the A431-6 and A431-7 subclones. Bars represent the mean ± SEM. in five independent experiments.
2. Western blotting images for pSTAT1 (Y701), pSTAT1 (S727), STAT1, and β-actin in A431-6 and A431-7 cells. Left: Second trial Right: Third trial
3. Western blotting results to validate the knockdown efficiency in siSTAT1#1 or siSTAT1#2-treated A431-6 cells.
4. Quantification of the cell cluster volume is shown in Fig. 2d. Lines show the mean with standard deviation (SD) for n=20 (siControl), n=18 (siSTAT1#1), and n=20 (siSTAT1#2) clusters in two independent experiments.
5. The sphericity of siControl-, siSTAT1 #1-, or STAT1 #2-treated A431-WT cell clusters were cultured in a three-dimensional collagen gel culture system. Lines show the mean with SD for n=22 (siControl), n=16 (siSTAT1#1), and n=22 (siSTAT1#2) clusters in two independent experiments.


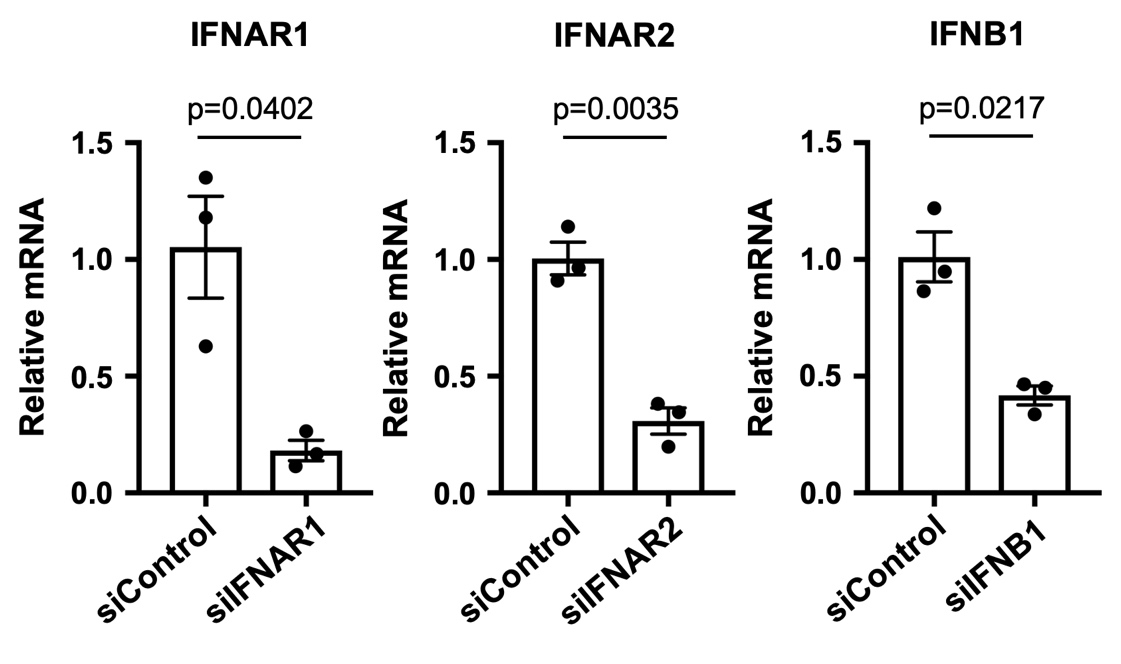


Supplementary Figure 3

qPCR results to validate the knockdown efficiency in siIFNAR1-, siIFNAR2- or siIFNB1-treated A431-6 cells. Bars represent mean ± SEM. in three independent experiments.


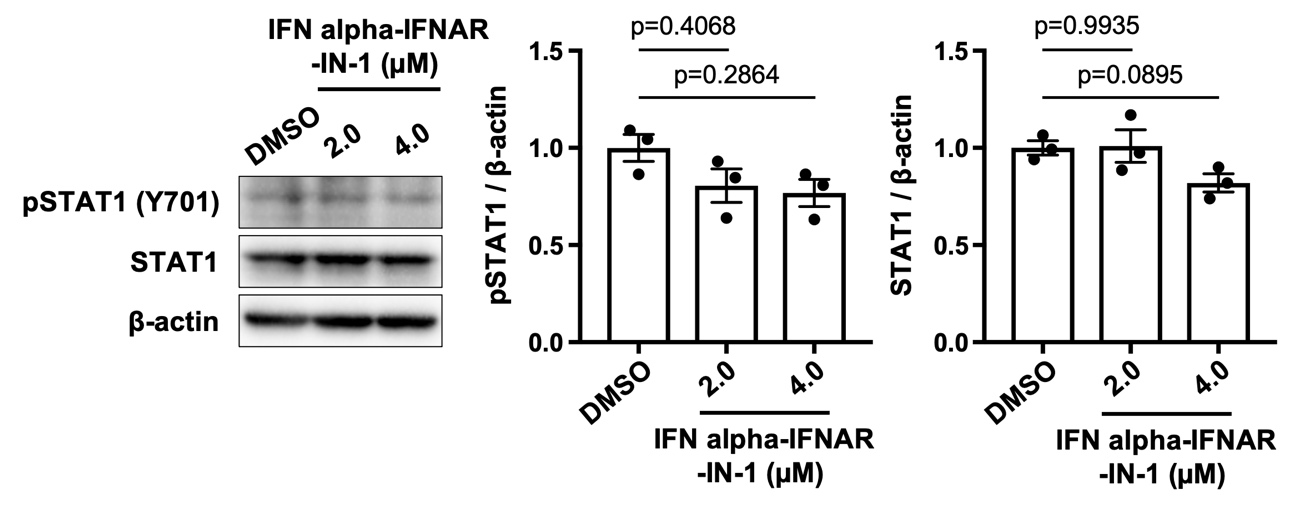


Supplementary Figure 4

Representative western blotting images for pSTAT1 (Y701), STAT1, and β-actin in DMSO, 2 µM- or 4 µM-IFN alpha-IFNAR-IN-1-treated A431-6 cells. Bars represent mean ± SEM. in three independent experiments.


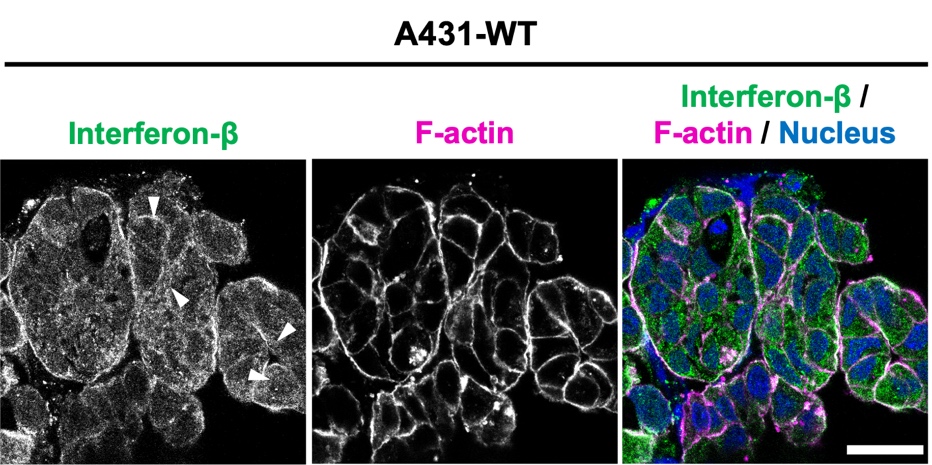


Supplementary Figure 5

Immunofluorescence images for interferon-β (INFB; green) with F-actin (magenta) and nucleus (blue) in A431-WT cell clusters in a three-dimensional collagen gel culture system. Arrowheads show immunoreactivity for IFNB in the intercellular spaces. Scale bars represent 25 µm.


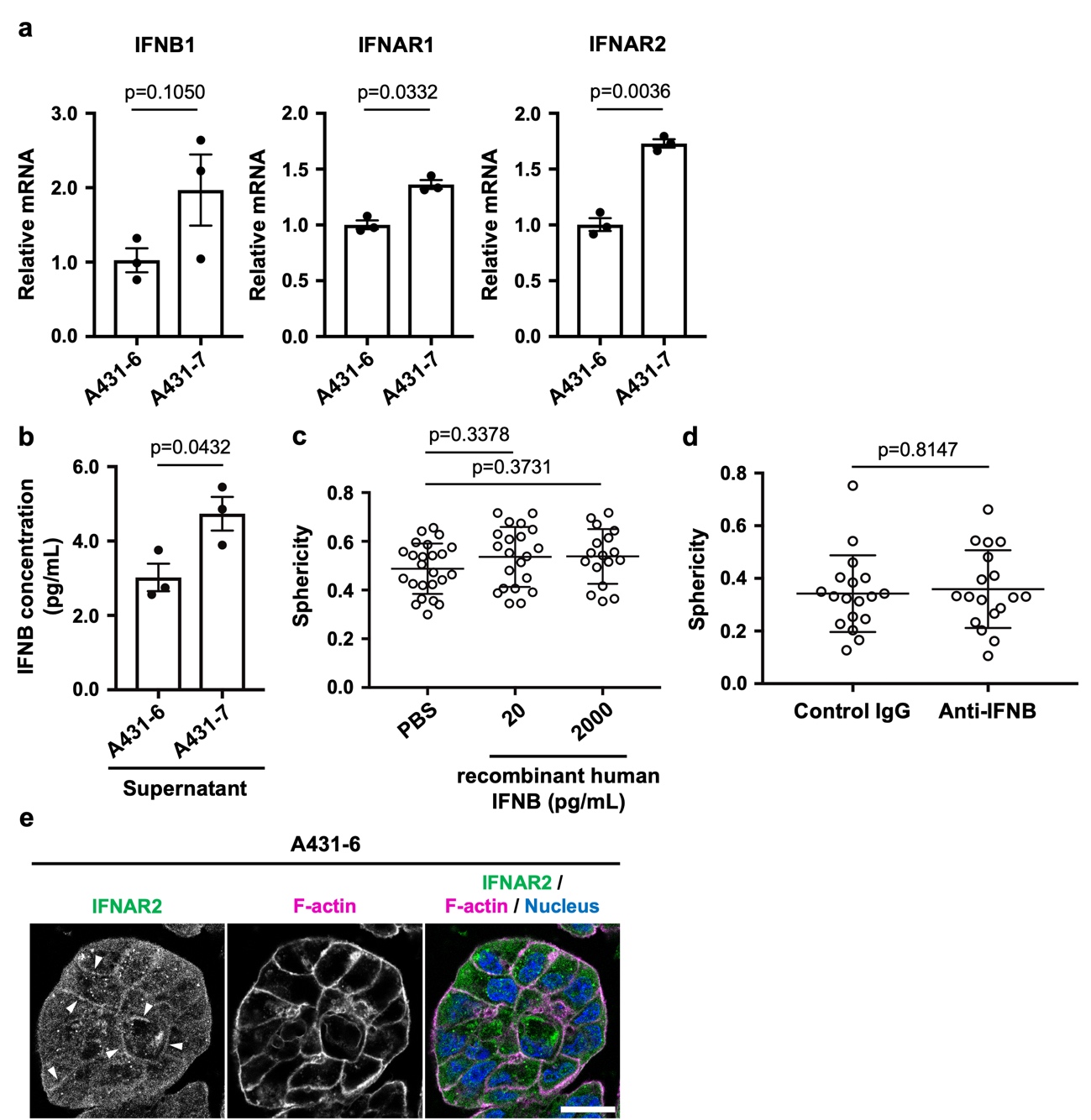


Supplementary Figure 6

1. qPCR results of IFNB1, IFNAR1, and IFNAR2 in A431-6 and A431-7 subclones. Bars represent mean ± SEM. in three independent experiments.
2. Measurement of IFNB concentration in culture supernatants of A431-6 and A431-7 cells by ELISA. Bars represent the mean ± SEM in three independent experiments.
3. The sphericity of 20 or 2000 pg/mL IFNB-treated A431-7 cell clusters was evaluated in a three-dimensional collagen gel culture system. Lines show the mean with SD for n=24 (PBS), n=21 (20 pg/mL), and n=17 (2000 pg/mL) clusters in two independent experiments.
4. The sphericity of 10 ng/mL IFNB neutralization antibody-treated A431-6 cell clusters cultured in a three-dimensional collagen gel culture system. Lines show the mean with SD in n=18 (control IgG) and n=18 (anti-IFNB) clusters from two independent experiments.
5. Immunofluorescence images of interferon receptor (IFNAR2; green) with F-actin (magenta) and nucleus (blue) in A431-6 cell clusters in a three-dimensional collagen gel culture system. Arrowheads show immunoreactivity for IFNB in intercellular spaces. Scale bars represent 20 µm.


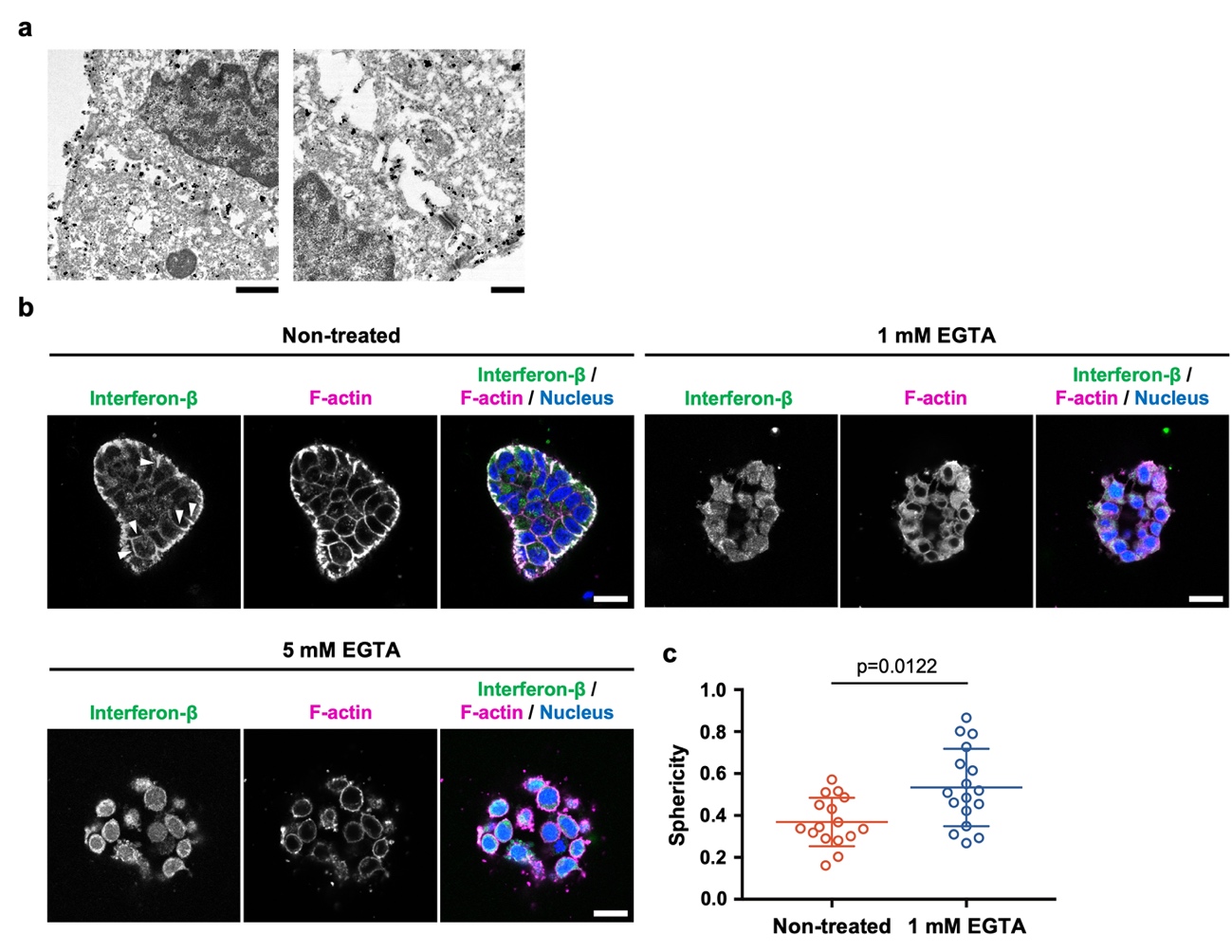


Supplementary Figure 7

1. Immunoelectron microscopy of IFNB. Black particles show localization of IFNB. Scale bars 500 nm.
2. Immunofluorescence images of IFNB (green) with F-actin (magenta) and nucleus (blue) in 1 or 5 mM EGTA-treated A431-6 cell clusters in a three-dimensional collagen gel culture system. Arrowheads show immunoreactivity for IFNB in intercellular spaces. Scale bars represent 25 µm.
3. The sphericity of 1 mM EGTA-treated A431-6 cell clusters cultured in a three-dimensional collagen gel culture system. Lines show the mean with SD for n=16 (Non-treated) and n=17 (1 mM EGTA) clusters by two independent experiments.


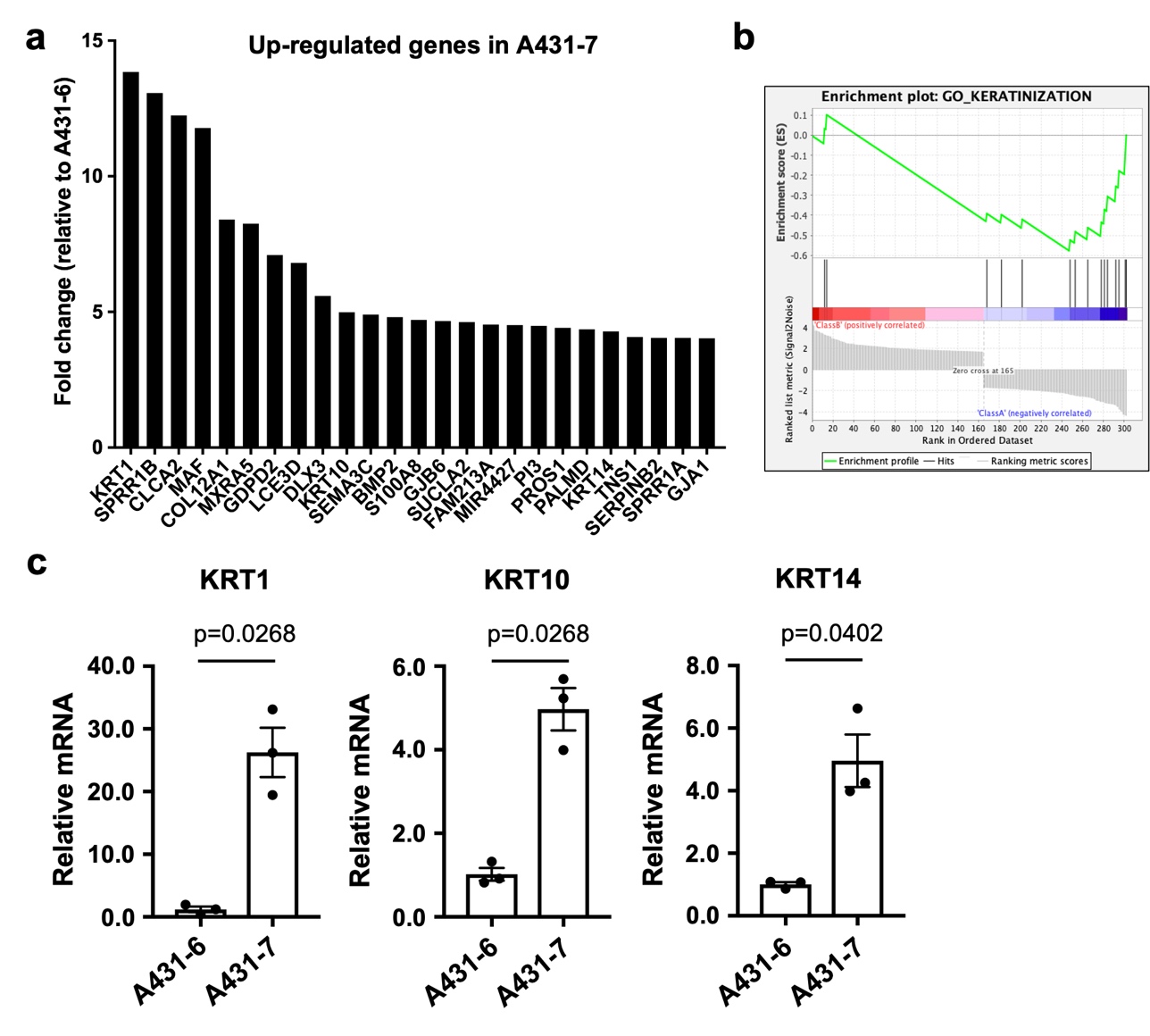


Supplementary Figure 8

1. The DNA microarray result comparing the A431-6 and A431-7 subclones. Genes with more than a 4-fold increase in A431-7 subclone are shown.
2. GSEA showing keratinization in A431-7 cells.
3. qPCR results of KRT1, KRT10 and KRT14 in A431-6 and A431-7 subclones. Bars represent mean ± SEM. in three independent experiments.

**
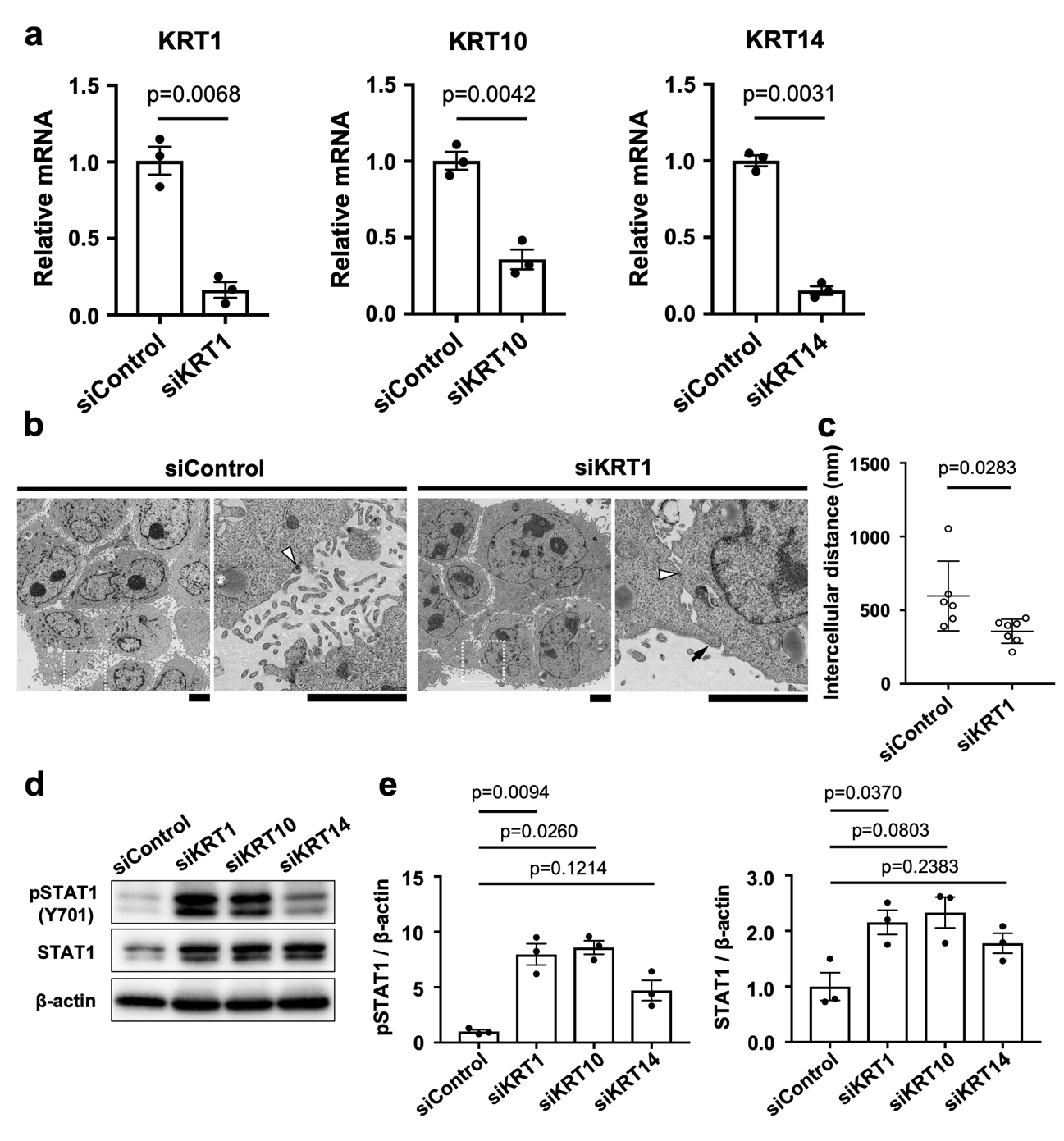
**

Supplementary Figure 9

1. qPCR results to validate the knockdown efficiency in siKRT1-, siKRT10-, or siKRT14-treated A431-7 cells. Bars represent mean ± SEM. in three independent experiments.
2. Representative transmission electron microscopic images of siControl- or siKRT1-treated A431-7 cell clusters cultured in a three-dimensional collagen gel culture system. The regions surrounded by white dotted squares are magnified on the right. The arrow indicates the sealed edge of the intercellular space. Arrowheads show intercellular junctions. Scale bars represent 3 µm.
3. Quantification of intercellular distances in I. Lines show the mean with SD in >5 clusters by two independent experiments.
4. Representative western blotting images for pSTAT1 (Y701), STAT1, and β-actin in siControl-, siKRT1-, siKRT10-, or siKRT14-treated A431-7 cells.
5. Quantification of protein bands in (e). Bars represent mean ± SEM. in three independent experiments.

**
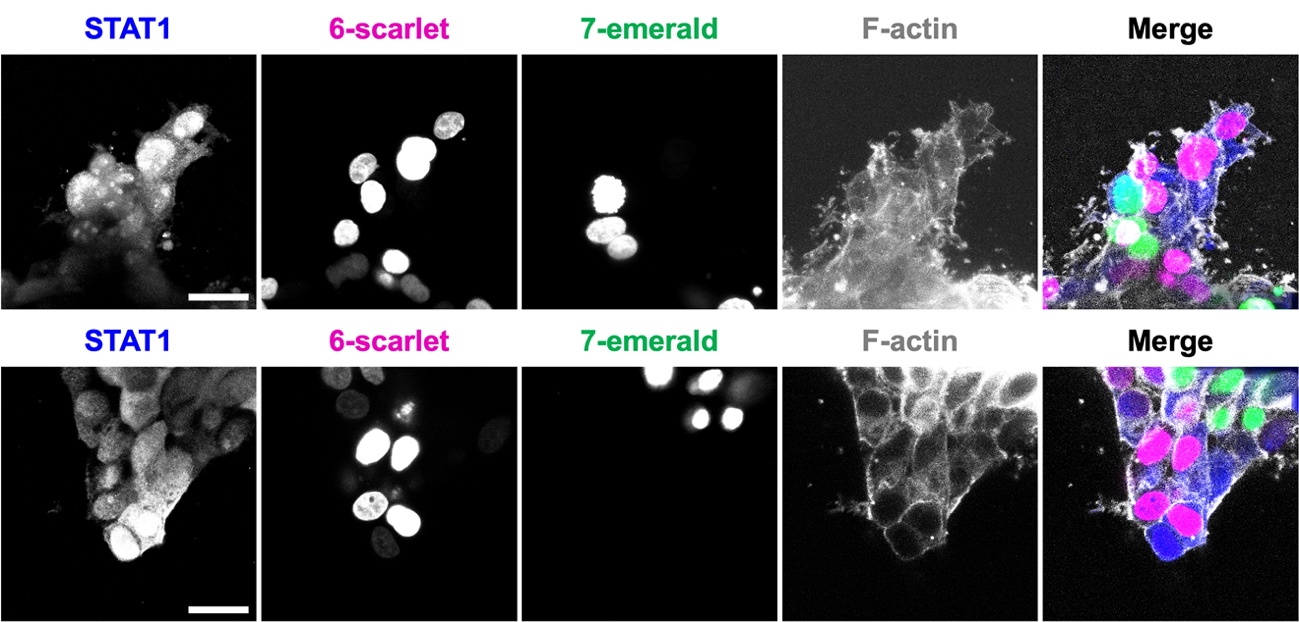
**

Supplementary Figure 10

Immunofluorescence images for STAT1 (blue) with F-actin (gray) in a mixed spheroid consisting of 6-scarlet (magenta) and 7-emerald (green) cells cultured in a collagen gel. Scale bars represent 25 µm.

**
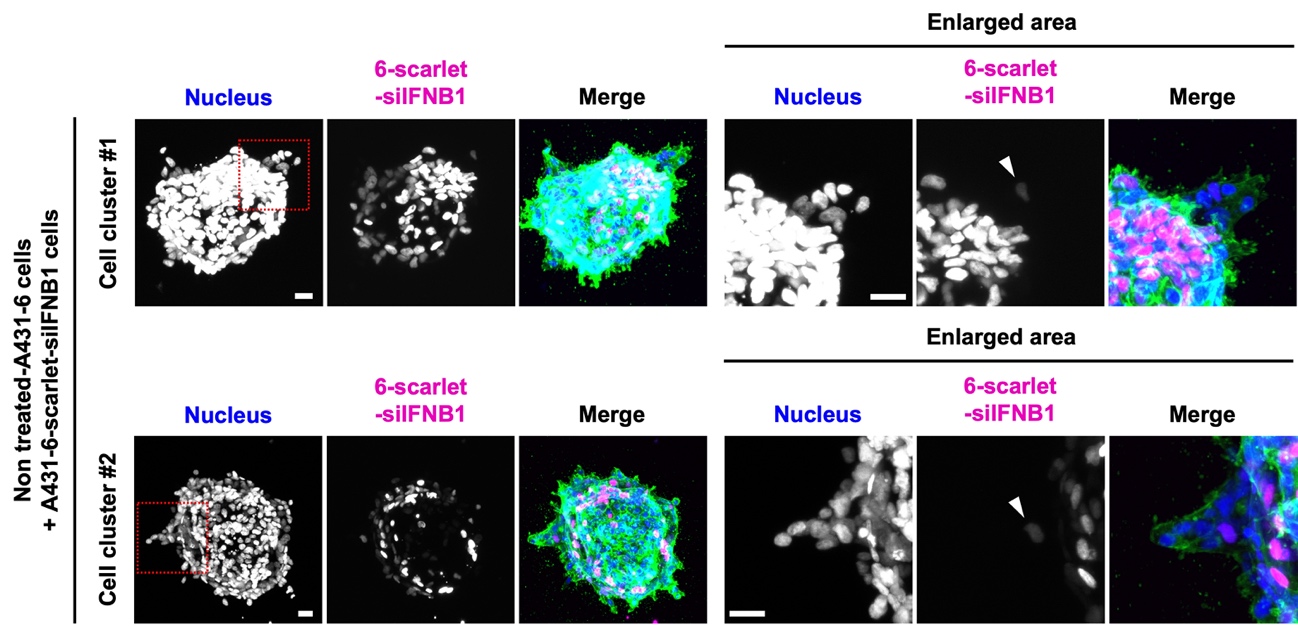
**

Supplementary Figure 11

1. The images showing the collective invasion of A431-6 cell cluster containing IFNB1-knockdown cells (6-scarlet-siIFNB, magenta). The region surrounded by red squares was enlarged in the right. Arrowheads show IFNB-knockdown cell in invasive protrusion. Blue: nucleus, Green: F-actin. Scale bar represents 30 µm.

**
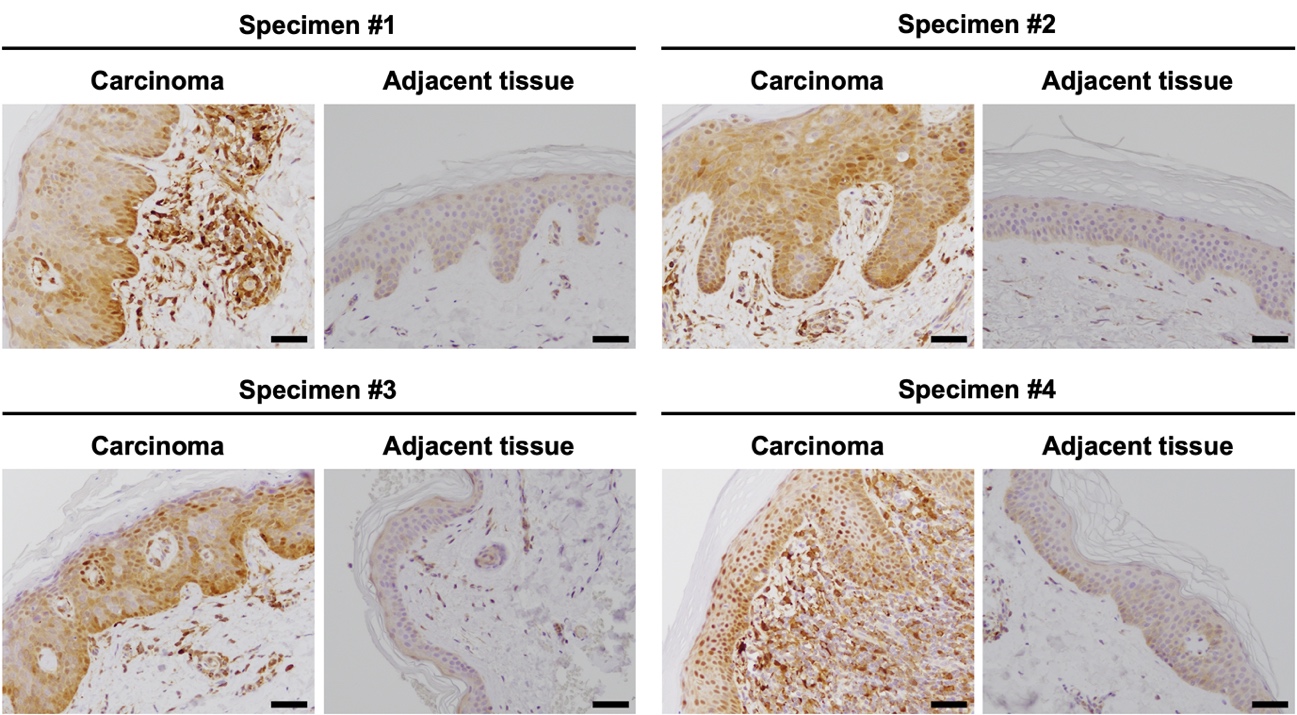
**

Supplementary Figure 12

Representative images of immunohistochemistry for STAT1 in human SCC *in situ* (Bo’en's disease). Carcinoma and adjacent tissue are a pair of the same specimens. The scale bars represent 50 µm.

**
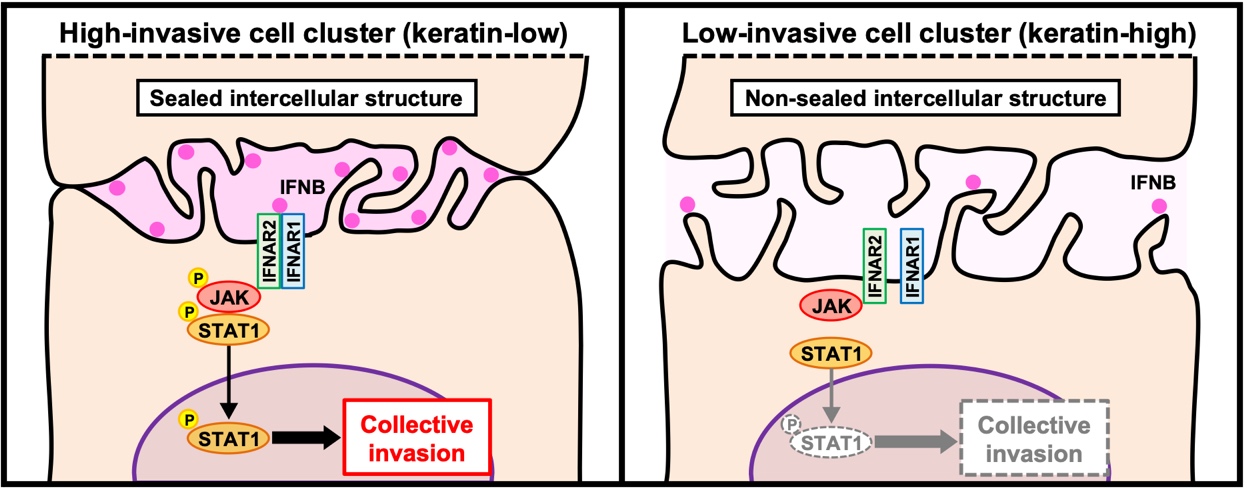
**

Supplementary Figure 13

Schematic illustration of the findings in this study. Interferon-β (IFNB) is localized to a sealed intercellular structure and drives collective invasion via activation of the type-I interferon receptor (IFNAR), Janus kinase (JAK), and signal transducer and activator of transcription 1 (STAT1) in the high-invasive cell clusters (left), whereas low-invasive cell clusters with high keratin expression possess non-sealed wider intercellular spaces, showing low STAT1 activity (right).

**Supplementary materials and methods**

**Three-dimensional collagen gel culture**

The cells were embedded into type-I collagen gels (Cell matrix I-P; Nitta Gelatin, Osaka, Japan) to assess their invasiveness. Cell suspensions were prepared at a concentration of 1 × 10^5^ cells/mL in 1.6 mg/mL collagen solution. This solution (600 µL) was added to a hand-made 16 mm inner diameter glass dish and incubated for 30 min at 37 °C to induce gelation. Assessment of the invasive potential and sphericity of A431 cell clusters was evaluated using this three-dimensional culture system.

**Fluorescent staining of cell clusters**

In fluorescent phalloidin staining, cell clusters in collagen gels were fixed with 4% paraformaldehyde (Nacalai Tesque, Kyoto, Japan) in phosphate buffered saline (PBS) for 2 h at 4 °C. Permeabilization and blocking were performed simultaneously using 1% bovine serum albumin (BSA) (Wako Pure Chemical Industries) and 0.3% Triton X-100 (Sigma-Aldrich Co. LLC) in PBS for 1 h at room temperature. Cells were incubated with Alexa Fluor-phalloidin 405 or 488 (Invitrogen, Carlsbad, CA, USA) diluted with Can Get Signal Immunostain (Immunoreaction Enhancer Solution B; TOYOBO, Osaka, Japan) overnight at 4 °C.

In immunofluorescent staining, the samples were prepared from the cells in collagen gels. Collagenase (0.1%) in PBS was added and incubated for 30 min at 37 °C. The cells were then fixed with 4% paraformaldehyde (Nacalai Tesque) in PBS for 1 h at 4 °C. Permeabilization and blocking were performed simultaneously using 1% BSA (Wako Pure Chemical Industries) and 0.3% Triton X-100 (Sigma-Aldrich Co. LLC) in PBS for 1 h at room temperature. Cells were incubated with a primary antibody diluted in Can Get Signal Immunostain (Immunoreaction Enhancer Solution B, TOYOBO) overnight at 4 °C. Next, the cells were incubated with a secondary antibody diluted with Can Get Signal Immunostain (Immunoreaction Enhancer Solution B, TOYOBO) and Alexa Fluor phalloidin 546 (Invitrogen) for 1 h at room temperature. Fluorescent images of phalloidin and immunofluorescent staining were captured from the top to the bottom of the cell cluster at 1-µm intervals by confocal laser microscopy (A1R or C2+ Confocal Imaging System; Nikon, Tokyo, Japan) equipped with a 20× (CFI Apo LWD Lambda S 20XC WI) or 60× objective lens (CFI Plan Apo VC 60xC WI) (Nikon).

**Morphological analysis using Imaris software**

3D images were constructed using Imaris software (Bitplane AG, Zürich, Switzerland). The sphericity (36πV^2^/S^3^) of cell clusters was calculated by the ratio of the cir’le's circumference (π), the surface area (S), and volume (V) of the clusters, as calculated by Imaris.

**Hanging drop method**

Spheroids used in Fig. 6 (6f, 6g, and 6h), Fig. S10, and Fig. S11 were prepared using the hanging drop method. Each droplet (10 µL) was seeded to contain 500 cells. The prepared spheroids were collected 2 days after seeding and embedded into 1.6 mg/mL collagen gels (Nitta Gelatin).

**Western blotting**

The samples were prepared from the cells in collagen gels. Cold 10% trichloroacetic acid (TCA) (Sigma-Aldrich Co. LLC) in PBS was added to the cells and incubated for 10 min at 4 °C. After rinsing three times with PBS to remove TCA, 0.1% collagenase (Wako Pure Chemical Industries) in PBS was added and incubated for 40 min at 37°C. After collagen digestion, collagenase was removed by centrifugation at 10,000 × *g* for 3 min. Proteins from the cells were extracted using sodium dodecyl sulfate (SDS) sample buffer (60 mM Tris HCl, 2.2% SDS, 10% glycerol, 100 mM dithiothreitol, pH 6.8) and boiled for 5 min. Western blotting was performed as previously reported ^30^. The primary antibodies were diluted to obtain signal solution 1 (TOYOBO). Horseradish peroxidase (HRP)-conjugated secondary antibodies were diluted to obtain signal solution 2 (TOYOBO). Protein signals were detected using an Immobilon Western Chemiluminescent HRP substrate (EMD Millipore, Billerica, MA, USA). Chemiluminescence signals were captured using a ChemiDoc Touch (Bio-Rad Laboratories, Richmond, CA, USA).

**Reagent treatment**

JAK inhibitor I (15 nM) (Sigma-Aldrich Co. LLC), IFN alpha-IFNAR-IN-1 (2.0 or 1.0 µM) (MedChemExpress, NJ, USA), recombinant human IFN-beta (2000 or 20 pg/mL) (R&D Systems, Inc., MN, USA), Anti IFN beta antibody (10 ng/mL) (NeutraKineTM, Proteintech Co., Manchester, UK), and EGTA (5 or 1 mM) (Sigma-Aldrich Co. LLC) were added to the A431-6 or A431-7 cells in a three-dimensional collagen gel culture system. All reagents were used at the stated concentrations, which were determined with reference to the reported IC50 and EC50 values.

**Small interfering RNA (siRNA) transfection**

Cells were seeded in 6-well plates at a density of 6 × 10^5^ cells/well and transfected with the appropriate siRNA using Lipofectamine RNAiMAX Reagent (Thermo Fisher Scientific, Inc. Waltham, MA, USA). Transfection was performed with siRNA concentrations of 15 or 2.5 nM. Nucleotide blasts demonstrated that there was no homologous sequence of control siRNA.

**Reverse transcription and quantitative PCR**

RNA from the cells cultured in collagen gels was extracted with Tripure reagent (Roche Applied Science, Indianapolis, IN, USA) and purified using the FastGene RNA Basic Kit (NIPPON Genetics Co., Ltd, Tokyo, Japan). cDNA was synthesized using ReverTra Ace (TOYOBO). Quantitative PCR (qPCR) was performed using KAPA SYBR FAST qPCR Kits (KK4602; NIPPON Genetics Co., Ltd) and StepOnePlus (Thermo Scientific) according to the manufacturer’s instructions. ACTB was used as an endogenous control.

**Enzyme-linked immunosorbent assay (ELISA)**

Interferon-β concentration in the culture medium was measured by sandwich ELISA using the Human IFN-beta DuoSet (R&D Systems, Inc.). Ninety-six-well microplates were coated with capture antibody diluted in PBS. The antibody-coated wells were blocked with 1.0% BSA in PBS, and then each sample was added. After washing three times with 0.05% Tween 20 containing PBS (TPBS), biotin-conjugated detection antibody and HRP-conjugated streptavidin were added. After washing three times with TPBS, TMB solution (Wako Pure Chemical Industries) was used as the chromogenic substrate. The absorbance at 450 nm was measured using a microplate reader (Bio-Rad).

**Transmission electron microscopy (TEM)**

The cells in collagen gels were treated with 0.1% collagenase in PBS for 30 min at 37 °C and then fixed with 2.5% glutaraldehyde (Nacalai Tesque) in 0.1 M phosphate buffer (pH 7.4) overnight at 4 °C. After postfixation with 1% OsO_4_ in ultrapure water for 90 min, the cells were embedded in a 2% agarose gel. The cells in agarose gels were dehydrated through a graded series of ethanol, and embedded in Epon resin (Quetol 812) according to a conventional method. Ultra-thin sections stained with uranyl acetate and lead citrate were observed under a transmission electron microscope (JEM1400, JEOL Ltd, Tokyo, Japan). The distance between two cells in the cell clusters was manually measured using the pencil tool (Fiji/ImageJ, National Institutes of Health, Bethesda, MD, USA).

**Immunoelectron microscopy**

Cells were processed using the pre-embedding silver-intensified immunogold method, as described in a previous study ^48^. Rabbit anti-IFN-β (Proteintech Co.) was used as the primary antibody. Nanogold-anti-rabbit IgG (1:200, Nanoprobes, Yaphank, NY, USA) was used as the secondary antibody. Gold signals were enhanced using HQ silver (nanoprobes). Observations were performed using a transmission electron microscope (JEM1400, JEOL Ltd.).

**Human samples of Bowen's disease**

Human samples of Bowen's disease were obtained at the time of surgery from patients who provided written informed consent at the Nagoya University Hospital. This study was conducted in accordance with the principles of the Declaration of Helsinki for Human Research and approved by the Ethics Committee of Nagoya University Graduate School of Medicine (approval no. 2017-0127-3).

**Statistical analysis**

Statistical significance was set at p < 0.05. All quantified data were graphed and tested using GraphPad Prism software (version 7.0; GraphPad Software Inc., San Diego, CA, USA). Statistical analysis was performed using Student’s t-test (ELISA and TEM), paired t-test (western blotting and qPCR in the two groups), and Dunnett’s multiple comparisons test (western blotting and qPCR with more than three groups). The sphericity of the cell clusters in the two groups was tested using the Mann-Whitney U test. For more than three groups, Dunn’s multiple comparison test was performed. The number of samples were recorded until the distribution of data was known. The test for normal distribution was performed using the Shapiro-Wilk test. The similarity of variances between the groups that are being compared was examined by F-test. All tests were two sided.

**Supplementary Information of reagents**

| **Antibodies** | | | |
| --- | --- | --- | --- |
| Products | Dilution rate | Source | Identifier |
| Rabbit anti-STAT1 | 1:2,000 | Cell Signaling Technology | 14994 |
| Rabbit anti-phospho-STAT1 (Tyr701) | 1:1,000 | Cell Signaling Technology | 9167 |
| Rabbit anti-phospho-STAT1 (Ser727) | 1:1,000 | Cell Signaling Technology | 9177 |
| Rabbit anti-phospho-JAK1 (Tyr1034/1035) | 1:1,000 | Cell Signaling Technology | 3331 |
| Mouse anti-beta actin | 1:100,000 | Abcam | ab6276 |
| Rabbit anti-IFN Beta | 1:500 | Proteintech | 27506-1-AP |
| Anti-Rabbit IgG, HRP-linked Antibody | 1:4,000 | Cell Signaling Technology | 7074 |
| Anti-Mouse IgG, HRP-linked Antibody | 1:100,000 | Cell Signaling Technology | 7076 |
| Goat anti-Rabbit IgG (H+L) Alexa Fluor 488 | 1:500 | Invitrogen | A27034 |

| **Phalloidins** | | | |
| --- | --- | --- | --- |
| Products | Dilution rate | Source | Identifier |
| Alexa Fluor 405 Phalloidin | 1:500 | Invitrogen | A30104 |
| Alexa Fluor 488 Phalloidin | 1:500 | Invitrogen | A12379 |
| Alexa Fluor 546 Phalloidin | 1:200 | Invitrogen | A22283 |

| **qPCR primers** | | |
| --- | --- | --- |
| Target genes | Forward / Reverse | Sequences (5’ to 3’) |
| OAS1 | Forward | GTGTCCAAGGTGGTAAAGGGTG |
|  | Reverse | AAGACAACCAGGTCAGCGTC |
| OAS2 | Forward | TGGCTCCTATGGACGGAAAAC |
|  | Reverse | AGGATGTCACGTTGGCTTCTC |
| IFI6 | Forward | TGCTGTGCCCATCTATCAGC |
|  | Reverse | TTTTTCTTACCTGCCTCCACCC |
| IFI44L | Forward | GGCCACCGTCAGTATTTGGAATG |
|  | Reverse | AGCCTATTTCTGTGCTCTCTGGC |
| IFNAR1 | Forward | AGCGATGAGTCTGTCGGGAATG |
|  | Reverse | GAGGACCAATCTGAGCTTTGCG |
| IFNAR2 | Forward | AGATGCTTTTGAGCCAGAATGCC |
|  | Reverse | ACACGAGGCTGATATACACCATGAG |
| IFNB1 | Forward | AAGCCTTTGCTCTGGCACAAC |
|  | Reverse | TGGAGAAGCACAACAGGAGAGC |
| KRT1 | Forward | GAGGGAGAAGAAAGCAGGATGTC |
|  | Reverse | ACTGATGGTGGTGTGGCTTG |
| KRT10 | Forward | ACCACGAGGAGGAAATGAAAGACC |
|  | Reverse | AACCAGGCTTCAGCATCTTTGC |
| KRT14 | Forward | AGCAGCAGAACCAGGAGTACAAG |
|  | Reverse | GAGGAGGTCACATCTCTGGATGAC |

| **siRNA** | | |
| --- | --- | --- |
| Target genes | Target sequences (5’ to 3’) |  |
| STAT1 #1 | AAGATGAATATGACTTCAAATGC |  |
| STAT1 #2 | TGACATCATTCGCAATTACAAAG |  |
| IFNAR1 | TGGCTTATAGTTGGAATTTGTAT |  |
| IFNAR2 | GTGGAAATTTCACCTATATCATT |  |
| IFNB1 | GGCTAATGTACTGCATATGAAAG |  |
| KRT1 | GAGAAATTCAAAGATAGAAATCC |  |
| KRT10 | TACAGAAATTGATAATAACATCC |  |
| KRT14 | ATCAATACAGCTTCATTATCTCC |  |
| Control (Guide) | AAACTACATGTCACATCACGG |  |
| Control (Passenger) | AACCGTGATGTGACATGTAGT |  |

**Supplementary dataset** (separate files). Raw results of DNA microarray in the A431-6 subclone versus the A431-7 subclone.
